# The potential for histone deacetylase (HDAC) inhibitors as cestocidal drugs

**DOI:** 10.1371/journal.pntd.0009226

**Published:** 2021-03-03

**Authors:** Hugo Rolando Vaca, Ana María Celentano, María Agustina Toscanini, Tino Heimburg, Ehab Ghazy, Patrik Zeyen, Alexander-Thomas Hauser, Guilherme Oliveira, María Celina Elissondo, Manfred Jung, Wolfgang Sippl, Federico Camicia, Mara Cecilia Rosenzvit

**Affiliations:** 1 Instituto de Microbiología y Parasitología Médica, Universidad de Buenos Aires-Consejo Nacional de Investigaciones Científicas y Tecnológicas (IMPaM, UBA-CONICET). Facultad de Medicina, Paraguay 2155, piso 13, C1121ABG, Buenos Aires, Argentina; 2 Laboratorio de Zoonosis Parasitarias, Instituto de Investigaciones en Producción, Sanidad y Ambiente (IIPROSAM), Facultad de Ciencias Exactas y Naturales (FCEyN), Universidad Nacional de Mar del Plata (UNMdP), Mar del Plata, Buenos Aires, Argentina; Consejo Nacional de Investigaciones Científicas y Técnicas (CONICET), Buenos Aires, Argentina; 3 Departamento de Microbiología, Parasitología e Inmunología, Facultad de Medicina, Universidad de Buenos Aires (UBA), Ciudad Autónoma de Buenos Aires, Argentina; 4 Institute of Pharmacy, Martin-Luther-University of Halle-Wittenberg, Halle(Saale), Germany; 5 Institute of Pharmaceutical Sciences, University of Freiburg, Freiburg, Germany; 6 Instituto Tecnológico Vale, Belém, Brazil; The University of Melbourne, AUSTRALIA

## Abstract

**Background:**

Echinococcosis and cysticercosis are neglected tropical diseases caused by cestode parasites (family Taeniidae). Not only there is a small number of approved anthelmintics for the treatment of these cestodiases, but also some of them are not highly effective against larval stages, such that identifying novel drug targets and their associated compounds is critical. Histone deacetylase (HDAC) enzymes are validated drug targets in cancers and other diseases, and have been gaining relevance for developing new potential anti-parasitic treatments in the last years. Here, we present the anthelmintic profile for a panel of recently developed HDAC inhibitors against the model cestode *Mesocestoides vogae* (syn. *M*. *corti*).

**Methodology/Principal findings:**

Phenotypic screening was performed on *M*. *vogae* by motility measurements and optical microscopic observations. Some HDAC inhibitors showed potent anthelmintic activities; three of them -entinostat, TH65, and TH92- had pronounced anthelmintic effects, reducing parasite viability by ~100% at concentrations of ≤ 20 μM. These compounds were selected for further characterization and showed anthelmintic effects in the micromolar range and in a time- and dose-dependent manner. Moreover, these compounds induced major alterations on the morphology and ultrastructural features of *M*. *vogae*. The potencies of these compounds were higher than albendazole and the anthelmintic effects were irreversible. Additionally, we evaluated pairwise drug combinations of these HDAC inhibitors and albendazole. The results suggested a positive interaction in the anthelmintic effect for individual pairs of compounds. Due to the maximum dose approved for entinostat, adjustments in the dose regime and/or combinations with currently-used anthelmintic drugs are needed, and the selectivity of TH65 and TH92 towards parasite targets should be assessed.

**Conclusion, significance:**

The results presented here suggest that HDAC inhibitors represent novel and potent drug candidates against cestodes and pave the way to understanding the roles of HDACs in these parasites.

## Author summary

Neglected tropical diseases, such as echinococcosis and cysticercosis, which are caused by taeniid cestodes (tapeworms), represent serious public health problems in many countries around the world. Given that there is only a small number of approved anthelmintics for the treatment of cestodiases, and that most of them are not highly effective against larval stages, identifying novel drug targets and their associated compounds is critical. Histone deacetylases (HDACs) are enzymes that produce epigenetic modifications of chromatin, thus modifying cellular gene expression. In this study, we evaluate and characterize a number of HDAC inhibitors on the model cestode *Mesocestoides vogae* and report the anthelmintic profile of these compounds. Some of the HDAC inhibitors tested showed potent anthelmintic effects, particularly entinostat, TH65 and TH92. These compounds were selected as the most promising candidates due to their high potencies, which were superior to the commercially-available anthelmintic drug albendazole. We also evaluated pairwise drug combinations of HDAC inhibitors and albendazole. The findings of this study provide a starting point for the development of new HDAC-based cestocidal compounds.

## Introduction

Human echinococcosis and cysticercosis are key zoonotic diseases caused by larval stages of tapeworm species of the genera *Echinococcus* and *Taenia* (family Taeniidae). Both echinococcosis and cysticercosis are recognized by the World Health Organization as neglected tropical diseases (NTDs) [1] and represent serious public health problems, disproportionately affecting socioeconomically disadvantaged populations around the world [2]. Common treatment against these diseases relies on a small number of approved cestocidal drugs, including the benzimidazole albendazole (ABZ) and the pyrazinoisoquinoline praziquantel (PZQ) [2, 3], which are not highly effective against human echinococcosis and cysticercosis. The precise mechanism of action of these compounds remains uncertain. However, it is known that ABZ blocks tubulin polymerization, impairs glucose uptake and leads to an uncoupling of oxidative phosphorylation. PZQ affects the parasites’ neuromusculature by disrupting Ca^2+^ homeostasis [3]. These cestocides are not well tolerated by some patients [4–6], ABZ, for instance, is ineffective in 40% of cystic echinococcosis cases [7, 8]. Thus, there is an imperative to search for new, safe and highly effective cestocidal compounds.

Targeting regulatory processes essential for parasite growth and development represents a promising starting point for identifying such candidates. In this context, chromatin-regulating enzymes (also known as chromatin regulators) make a series of covalent modifications in DNA, histone tails and other cellular effectors that alter chromatin structure. These enzymes regulate gene transcription and other essential processes for parasite survival and development [9, 10]. Moreover, enzymes involved in chromatin-regulating processes, such as DNA methyltransferases and those associated with histone post-translational modifications (particularly acetylation/deacetylation of lysine) have been extensively studied as drug targets for various diseases [11–13]. Histone acetylation is a reversible process, relying on the opposing effects of histone acetyltransferase and deacetylase enzymes. These enzymes catalyze the addition or removal of an acetyl group on the lysine residues of histone tails and non-histone proteins, which directly influences the chromatin structure, thus regulating gene transcription and other cellular processes. Histone deacetylase (HDAC) enzymes have been widely studied and validated as drug targets in diseases such as cancer, diabetes, autoimmune, and neurological disorders [11–13]. Recently, HDACs inhibition has also shown to affect parasitic diseases, such as schistosomiasis, leishmaniasis, Chagas and malaria [14–16]. Drug repurposing represents an excellent strategy for identifying new uses for approved or investigational drugs outside the original medical indication [17, 18], and, in this context, promising results have been achieved for the treatment of diseases caused by protozoa and trematodes with clinically approved HDAC inhibitors used against cancer [16, 19–22]. In trematodes, HDACs have been well characterized and validated as putative drug targets in *Schistosoma mansoni* [23–25]. In this parasite, HDACs have shown to play an important role in differentiation and reproduction as well as the interaction with the host in studies through the suppressing of specific genes by interfering RNA [24–26]. In relation to cestodes, available information about HDAC enzymes and inhibitors is scarce.

Recently, we have identified and characterized various Class I- and Class II-HDAC coding genes in some cestodes, and analyzed their transcriptional expression levels throughout several developmental stages of *Echinococcus* spp [27]. Furthermore, the inhibition of these enzymes by the pan-HDAC inhibitor trichostatin A (TSA) decreased parasite viability and induced alterations on the tegument and parasite structures (morphology) more generally. TSA also increased acetylation levels of total proteins, including histone H4, on the model cestode *Mesocestoides vogae* (syn. *M*. *corti*) [27]. These results suggested that HDACs represent potential drug targets in cestodes. In particular, it was observed by homology-modeling that the isotype HDAC8 from cestodes displays particular structural features that distinguish them from its homolog in *Homo sapiens* [27, 28]. These characteristics are similar to the previously reported for the crystal structure of HDAC8 from *S*. *mansoni* [26] and have been applied to the structure-based design of selective inhibitors. In this way, a series of mercaptoacetamide [29], benzhydroxamate [30, 31], cinnamic acid [32] and triazole [33] derivatives were synthesized, among others. Several of these compounds have shown high selective activities for HDAC8 from *S*. *mansoni* (SmHDAC8) or *Homo sapiens* (HsHDAC8) over the major human HDAC isoforms (HDAC1 and HDAC6), displaying low nanomolar activity against both HDAC8s. A number of these compounds are more active on SmHDAC8 than on HsHDAC8. Furthermore, several SmHDAC8 inhibitors have shown to be effective against both adult and larval schistosomes, and have displayed limited toxicity to human cell lines [29–33]. The conservation of these characteristics in cestode HDAC8s suggests that selective SmHDAC8 inhibitors should be active against cestode HDAC8s and potentially novel anthelmintic drugs against these parasites. Thus, studying the effect of HDAC inhibitors against cestodes represents a promising avenue toward developing novel cestocidal compounds.

This study aimed to determine and characterize the anthelmintic activity profile of several Class I- and Class II-HDAC inhibitors, including both clinically approved compounds and recently-developed selective HDAC inhibitors, using *M*. *vogae* larval stage as a cestode model. We also aimed to study the pharmacological potential of several pairwise drug combinations of the most promising HDAC inhibitors and the current anthelmintic drug ABZ. The evaluation and characterization of various inhibitors against HDAC enzymes should help explore the roles of these enzymes in cestodes, to aid in the development of new cestocidals.

## Materials and methods

### Ethics statement

Experiments involving the use of experimental animals were conducted strictly in accordance with the protocols approved by the Comité Institucional para el Cuidado y Uso de Animales de Laboratorio (CICUAL), Facultad de Medicina, Universidad de Buenos Aires (UBA), Argentina (protocols “*in vivo* passages of cestode parasites from *Mesocestoides vogae*” number CD N 1127/2015 and 1229/2015).

### Parasite material

The larval stages -tetrathyridia (TTy)- of *M*. *vogae* [34, 35] were maintained in the laboratory by alternate intraperitoneal infection of adult Wistar female rats (3 months old) and adult Balb/c female mice (3 months old), as described previously [36]. The experimental animals were bred and housed in a temperature-controlled light cycle room with food and water *ad libitum* at the animal facilities of the Instituto de Investigaciones en Microbiología y Parasitología Médica (IMPaM), Facultad de Medicina, Universidad de Buenos Aires (UBA)-Consejo Nacional de Investigaciones Científicas y Tecnológicas (CONICET), Ciudad Autónoma de Buenos Aires, Argentina. After three months of infection, mice were sacrificed by CO_2_ inhalation. TTy were collected from the peritoneal cavity using standard aseptic techniques and washed three times with sterile phosphate-buffered saline solution (PBS) pH 7.2 with levofloxacin (20 μg/mL).

Before being employed in experiments, TTy were size-selected using monofilament polyester meshes to a final size of 150 to 250 μm and incubated for 24 h in 5 mL of MvRPMI medium -a modified RPMI 1640 medium without phenol red (Sigma-Aldrich, USA) complemented with 10% v/v inactivated fetal bovine serum (INTERNEGOCIOS SA, Argentina), 2.4 g/L HEPES Free acid (JT Baker, USA), 2.5 g/L glucose (4.5 g/L final concentration, Britania, Argentina), 2 g/L Sodium Bicarbonate (Anedra, Argentina), 20 μg/mL levofloxacin (Tavanic, SANOFI, Argentina) and 1% v/v Pen/Strep (Penicillin-Streptomycin 10,000 U/mL, Gibco, USA)- at 37°C under 5% CO_2_ atmosphere.

### Compounds

The HDAC inhibitors used in this work are shown in S1 Table. Compounds of the series TH and TB were synthesized and purified as described previously [30–32]. TH119, TH138, TH139, EG13, EG18 and EG20 were synthesized and purified as described in the supplementary methodology (S1 Text). TSA was purchased from Cell Signaling Technology (USA). PZQ and ABZ were purchased from Sigma-Aldrich (USA). All stock solutions were prepared at 10 mM in 100% dimethylsulfoxide (DMSO) and stored at -20°C until use.

### Anthelmintic testing *in vitro*

The effect of each compound on parasite viability was determined using a motility assay employing a worm tracker device (WMicrotracker Designplus SRL, Argentina) [37], previously adapted to measure the movement of *M*. *vogae* TTy [27]. Briefly, 5 TTy were incubated in U-shape 96-well microplates (Greiner Bio-One, Germany) with 200 μL of MvRPMI medium per well at 37°C under 5% CO_2_ atmosphere up to 9 days, without changing the medium. Compounds were tested at concentrations of 2, 20 and 50 μM. Parasites pre-treated with ethanol 70% for 30 min were used as a positive control. PZQ, ABZ, and TSA at 20 μM were also evaluated to be used as additional positive controls. All motility assays were performed using an equal amount of the drug vehicle (1% DMSO final concentration) and corresponding negative controls (1% DMSO). Furthermore, to determine any possible morphological alterations on parasites treated with the compounds, parasite cultures were inspected daily. Images were taken using an inverted microscope (Primo Vert, Carl Zeiss, Germany) coupled to a digital video camera (AxioCam ERc5c, Carl Zeiss, Germany).

Phenotypic screening data were collected from three independent biological replicates, each corresponding to TTy obtained from a different mouse, in quadruplicate for each condition. Relative motility indices were determined as described previously [27, 38]. Statistical analyses were carried out using GraphPad Prism 8.0.2. Two-way ANOVA tests were used to analyze the effect of the compounds on *M*. *vogae* TTy viability. Significant differences (P < 0.05) were determined by Dunnett’s comparisons post-tests, comparing each compound concentration with the negative control (each run on each day).

### Evaluation of the dose-dependent effect

The dose-dependent *in vitro* effect of selected compounds on parasite viability was evaluated using the *M*. *vogae* TTy motility assay, as described above. Briefly, selected HDAC inhibitors and ABZ were tested at concentrations ranging from 2 μM (or 0.01 μM for entinostat) to 50 μM (or 20 μM for TH65). Relative motility indices were determined after 6 days of treatment from three independent biological replicates, each corresponding to TTy obtained from a different mouse, in quadruplicate for each condition. The half-maximal (IC_50_), 90% (IC_90_), and 25% (IC_25_) inhibitory concentration values were determined from dose-dependent curves generated by non-linear regression analysis using GraphPad Prism 8.0.2. One-way ANOVA tests were used for statistical analysis of differences in IC_50_ values. Significant differences (P < 0.05) were determined by Dunnett’s comparisons post-tests, comparing the IC_50_ determined for each HDAC inhibitor with the determined for ABZ.

### Evaluation of irreversibility of the anthelmintic *in vitro* effect

The irreversibility of the anthelmintic *in vitro* effect of selected HDAC inhibitors and ABZ was determined using the *M*. *vogae* TTy motility assay (described above). Briefly, TTy were incubated with the compounds at their respective IC_90_ concentrations for 6 days; then, culture medium was removed and TTy were gently washed four times in PBS (pH 7.2) at 37°C. Finally, TTy were incubated for 8 additional days in a fresh culture medium without adding the compounds. Relative motility indices were determined from three independent biological replicates, each corresponding to TTy obtained from a different mouse, in quadruplicate for each condition.

### Ultrastructural studies by scanning electron microscopy

Samples of *M*. *vogae* TTy were processed for Scanning Electron Microscope (SEM) to determine the effect of selected HDAC inhibitors on the tegument and parasite morphology at the ultrastructural level, as previously described [39]. Briefly, TTy were incubated with compounds at 20 μM for 6 days, then washed four times in PBS (pH 7.2) and fixed with 3% glutaraldehyde in phosphate buffer 0.1 M pH 7.4 (PB) for 72 h at 4°C. The samples were then washed four times in PB and dehydrated by sequential incubations in increasing concentrations of ethanol (50–100%). Finally, parasites were immersed in hexamethyldisilazane for 5 min, 1 h, and overnight and then sputter-coated with gold (100 Å thick). Parasites incubated with 1% DMSO were used as a negative control. The samples were inspected, and images were taken using a JEOL JSM-6460 LV scanning electron microscope operating at 15 kV.

### Testing of pairwise drug combinations

To assess the effect on parasite viability of all pairwise drug combinations of selected HDAC inhibitors and ABZ, a simplified and modified protocol of Planer et al. [40] was used. Briefly, the compounds were evaluated individually and in a pairwise manner with ABZ, testing each compound at its respective IC_25_ concentration using the *M*. *vogae* TTy motility assay (described above). Relative motility indices were measured up to 9 days of treatment from three independent biological replicates, each corresponding to TTy obtained from individual mice, in quadruplicate for each condition. Relative motility indices measured for each binary drug combination (measured effect of the pair) was compared to the predicted effect of the combination as follows: The predicted effect of the combination was expected to be the product of the relative motility indices determined for each compound when they were tested alone in a simple additive effect model [40]. Proportional effect indices were calculated based on the following equation:

$$Proportional\;effect\;index=\frac{Predicted\;effect\;of\;pair-Measured\;effect\;of\;pair}{Predicted\;effect\;of\;pair}$$

## Results

### Albendazole, praziquantel, and trichostatin A affect *Mesocestoides vogae* viability and are useful as control drugs

The effects of the anthelmintic drugs PZQ and ABZ, and the pan-HDAC inhibitor TSA at 20 μM provided initial parameters for the *M*. *vogae* TTy viability determination (motility assay and visual inspections). First, we confirmed that the vehicle used to dissolve drugs (DMSO) had no effect on parasite viability throughout duration of the assay. DMSO at 1% did not significantly affect parasite viability, even up to 9 days of incubation (S1 Fig), suggesting that DMSO at 1% is not toxic to *M*. *vogae* TTy and could be used as a drug vehicle. Then, the anthelmintic effects of PZQ, ABZ, and TSA were evaluated using the *M*. *vogae* TTy motility assay. A significant reduction on parasite viability was observed after 1 (55.0%; p < 0.0001), 1 (24.8%; p < 0.0001) and 5 (57.1%; p < 0.0001) days of treatment with PZQ, ABZ, and TSA, respectively (S2 Fig). Particularly, PZQ was able to kill all TTy (reducing parasite viability by 100%) at ≥ 4 days of treatment. Visual inspection revealed that PZQ, ABZ and TSA induced extensive damage to the tegument, with general morphological alterations after 1, 2, and 6 days of treatment, respectively, compared with untreated TTy (S3 Fig). Tegument debris could be observed in the culture medium as well as the influx of culture medium into the TTy. Both methods used for parasite viability determination showed anthelmintic effects of the compounds in a time-dependent manner. Additionally, a correlation between the intensity of morphological damage and parasite viability, determined using the *M*. *vogae* TTy motility assay, was observed.

After setting up *M*. *vogae* TTy viability determination methods, phenotypic screening was carried out to determine the anthelmintic profile of 20 HDAC inhibitors *in vitro*. Initially, four compounds against Class I HDACs and four against Class II HDACs were evaluated. Based on their anthelmintic effect, some structural analogs were also included in the analysis.

### Class I-HDAC inhibitors showed a potent anthelmintic effect against *Mesocestoides vogae*

**Fig 1:**
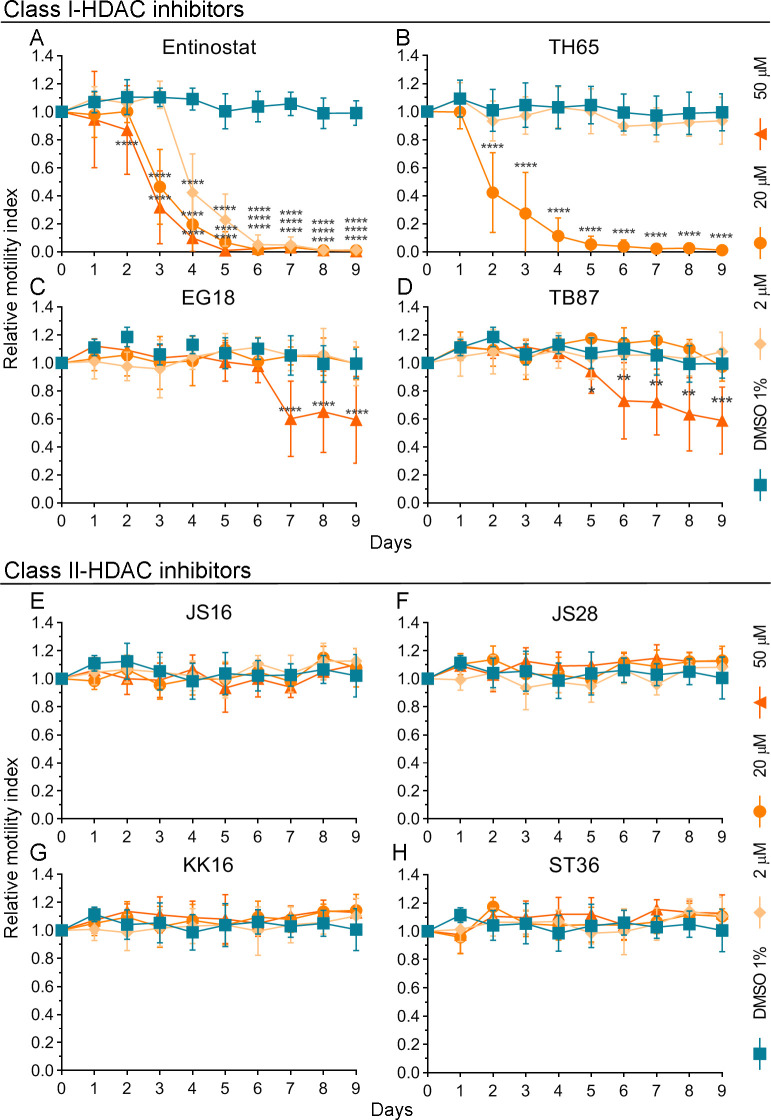
Effect of Class I- and Class II-HDAC inhibitors on *Mesocestoides vogae* TTy (tetrathyridia) viability. *In vitro* anthelmintic activity was determined for the Class I-HDAC inhibitors: (A) entinostat, (B) TH65, (C) EG18, and (D) TB87; and the Class II-HDAC inhibitors: (E) JS16, (F) JS28, (G) KK16, and (H) ST36. The compounds were evaluated at concentrations of 2, 20 and 50 μM (TH65 was not evaluated at 50 μM due to low solubility) and at different incubation times, using the *M*. *vogae* TTy motility assay. Parasites incubated with the drug vehicle (DMSO 1%) were used as a negative control. Relative motility indices were measured from three independent biological replicates, each one in quadruplicate. Error bars represent the standard deviation and the asterisks indicate those values that showed differences with statistical significance compared to the negative control, according to two-way ANOVA test and Dunnett’s post-tests (*, p < 0.05; **, p < 0.01; ***, p < 0.001; ****, p < 0.0001).

The anthelmintic effect of the Class I-HDAC inhibitors entinostat, TH65, EG18, and TB87 was determined *in vitro* using the *M*. *vogae* TTy motility assay (Fig 1A–1D; respectively). Entinostat and TH65 were the most potent Class I-HDAC inhibitors, showing a strong anthelmintic effect, in a time and dose-dependent manner (Fig 1A and 1B, respectively). Entinostat (also known as SNDX-275 and MS-275) is a benzamide group-containing HDAC inhibitor, selective against Class I-HDAC enzymes [41]. This compound showed a significant reduction on TTy viability after 2 days of treatment at 50 μM (12.9%; p < 0.0001), and after 3 and 4 days at 20 μM (53.6%; p < 0.0001) and 2 μM (57.7%; p < 0.0001), respectively (Fig 1A). Entinostat was also able to kill all TTy after 5 days of treatment at 50 μM and 6 days at 2 μM and 20 μM. Furthermore, extensive damage on the tegument and other morphological alterations were observed after 4 days of treatment with entinostat at 20 μM and 50 μM and after 6 days at 2 μM; compared to untreated-parasites (S4 Fig). The main change observed was an elongation of the body. The TTy appeared elongated and flattened. The presence of some constrictions on the body could be observed as well as an influx of culture medium into the worms and blebs on the tegument. In addition, tegumental debris was observed in the culture medium. Similarly, the effects of the structure-based designed SmHDAC8 inhibitors TH65, EG18, and TB87 [30, 32, 42] on *M*. *vogae* viability were determined. TH65 showed a significant reduction on *M*. *vogae* TTy viability after 2 days of treatment at 20 μM (57.9%; p < 0.0001) and was also able to kill all TTy after 5 days of treatment at 20 μM (Fig 1B). Major alterations on the tegument, leading to morphological disintegration, were observed in treated-parasites after 2 days of treatment at 20 μM (S5 Fig). Although we intended to evaluate TH65 at 50 μM, some crystal-like structures were observed in the culture medium with TH65 at 50 μM after 1 day of treatment (S5 Fig). This finding suggested limited solubility of TH65 and implied that 50 μM was not the actual concentration reached. For this reason, we did not include the measurements obtained for TH65 at 50 μM in the analysis. Furthermore, EG18 and TB87 were each less potent than each entinostat and TH65. Neither of these two compounds could kill all TTy at any tested concentrations, even up to 9 days of treatment (Fig 1C and 1D, respectively).

### Class II-HDAC inhibitors did not show a significant anthelmintic effect against *Mesocestoides vogae*

The effects on parasite viability of the Class II-HDAC inhibitors JS16, JS28, KK16, and ST36, were also evaluated using the *M*. *vogae* TTy motility assay (Fig 1E–1H; respectively). No significant effect on parasite viability and morphology was observed for these compounds at any tested concentrations, even up to 9 days of treatment compared to untreated-parasites.

### SmHDAC8 structure-based designed inhibitors showed a potent anthelmintic effect against *Mesocestoides vogae*

**Fig 2:**
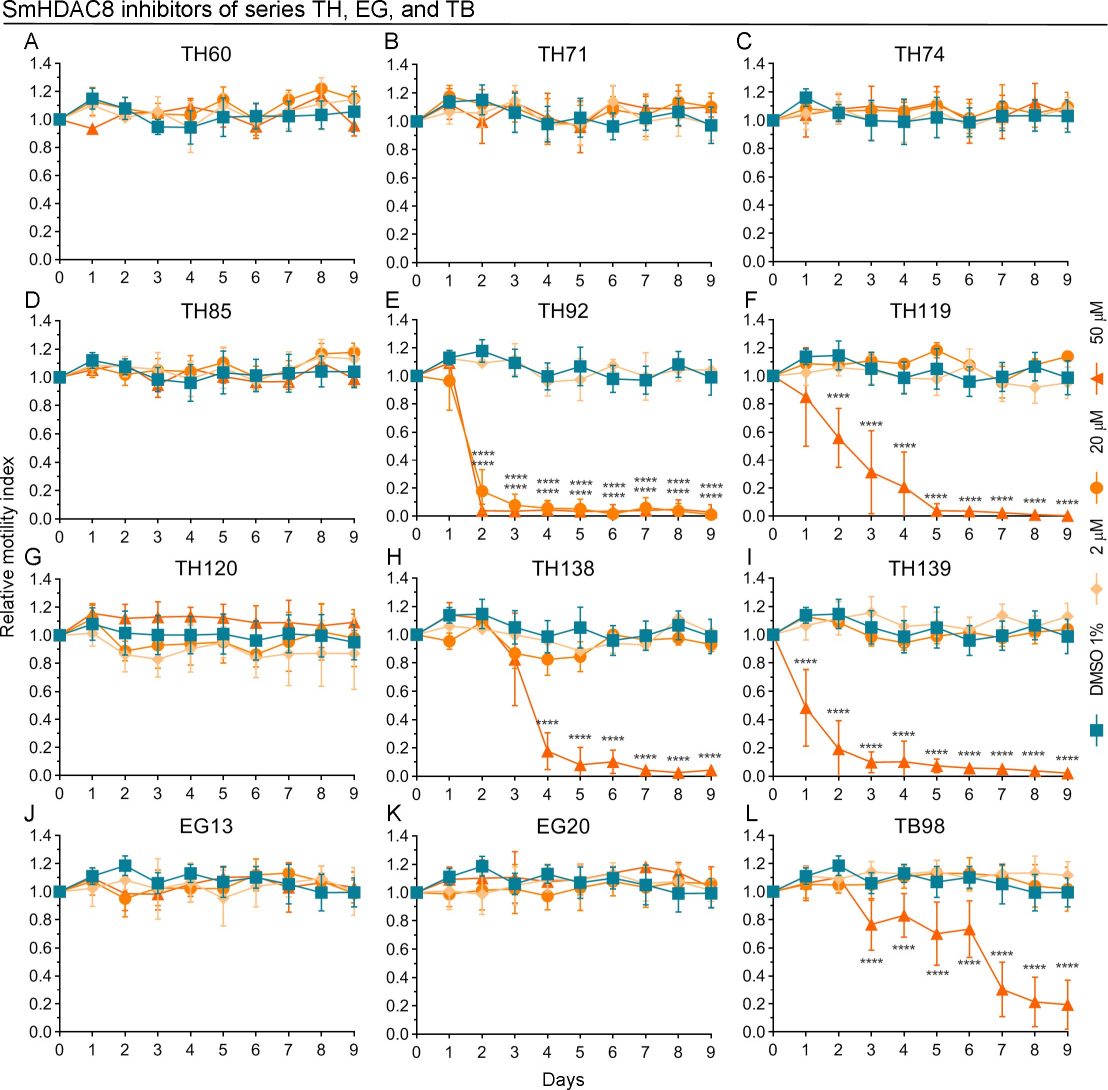
Effect of SmHDAC8 inhibitors (series TH, EG, and TB) on *Mesocestoides vogae* viability. *In vitro* anthelmintic activity was determined for compounds of the series TH: (A) TH60, (B) TH71, (C) TH74, (D) TH85, (E) TH92, (F) TH119, (G) TH120, (H) TH138, and (I) TH139; series EG: (J) EG13 and (K) EG20; and series TB: (L) TB98. All compounds were evaluated at concentrations of 2, 20, and 50 μM and at different incubation times using the *M*. *vogae* TTy motility assay. Parasites incubated with the drug vehicle (1% DMSO) were used as a negative control. Relative motility indices were measured from three independent biological replicates, each one in quadruplicate. Error bars represent the standard deviation and the asterisks indicate those values that showed differences with statistical significance compared to the negative control, according to two-way ANOVA test and Dunnett’s post-tests (*, p < 0.05; **, p < 0.01; ***, p < 0.001; ****, p < 0.0001).

Due to the potent anthelmintic effect showed by the SmHDAC8 inhibitors initially tested (Fig 1), various structural analogs of these compounds, belonging to the TH, EG and TB series, were also evaluated (Fig 2). The compounds of the series TH and EG are benzhydroxamate derivatives that showed activities in the nanomolar range against SmHDAC8 [30, 31, 42]. Several of these compounds showed a pronounced anthelmintic effect against *M*. *vogae* (Fig 2). In particular, TH92 showed a significant reduction on *M*. *vogae* TTy viability after 2 days of treatment at 20 μM (82.2%; p < 0.0001) and 50 μM (96.0%; p < 0.0001), being also able to kill all TTy after 3 days of treatment at 20 μM and 50 μM (Fig 2E). TH92 also induced a major alteration to the tegument, with a total loss of morphological integrity at 20 μM and 50 μM after 2 days of treatment (S6 Fig). The other compounds of the series TH were less potent than TH92. Only the structurally related compounds TH119, TH138, and TH139, were able to kill all TTy at 50 μM after 5, 7, and 6 days of treatment, respectively (Fig 2F, 2H and 2I; respectively). Furthermore, some alterations on the tegument and morphology were observed on TTy treated with several compounds of the series TH. Concerning the TB series, these compounds are cinnamic acid derivatives that showed activities in the nanomolar range against SmHDAC8 [32]. TB98 showed a significant reduction (23.3%; p < 0.0001) on *M*. *vogae* TTy viability at 50 μM after 3 days of treatment (Fig 2L). Finally, none of the EG series compounds showed a significant effect on *M*. *vogae* TTy viability at any of the concentrations tested, even up to 9 days of treatment.

### Selected HDAC inhibitors had dose-dependent anthelmintic effects *in vitro* -which were irreversible- with higher potencies than albendazole

The Class I-HDAC inhibitors entinostat, TH65, and TH92 were selected for further characterization based on their potent anthelmintic effect, as they were able to kill all TTy at 20 μM concentration before 9 days of treatment (see Figs 1 and 2).

**Fig 3:**
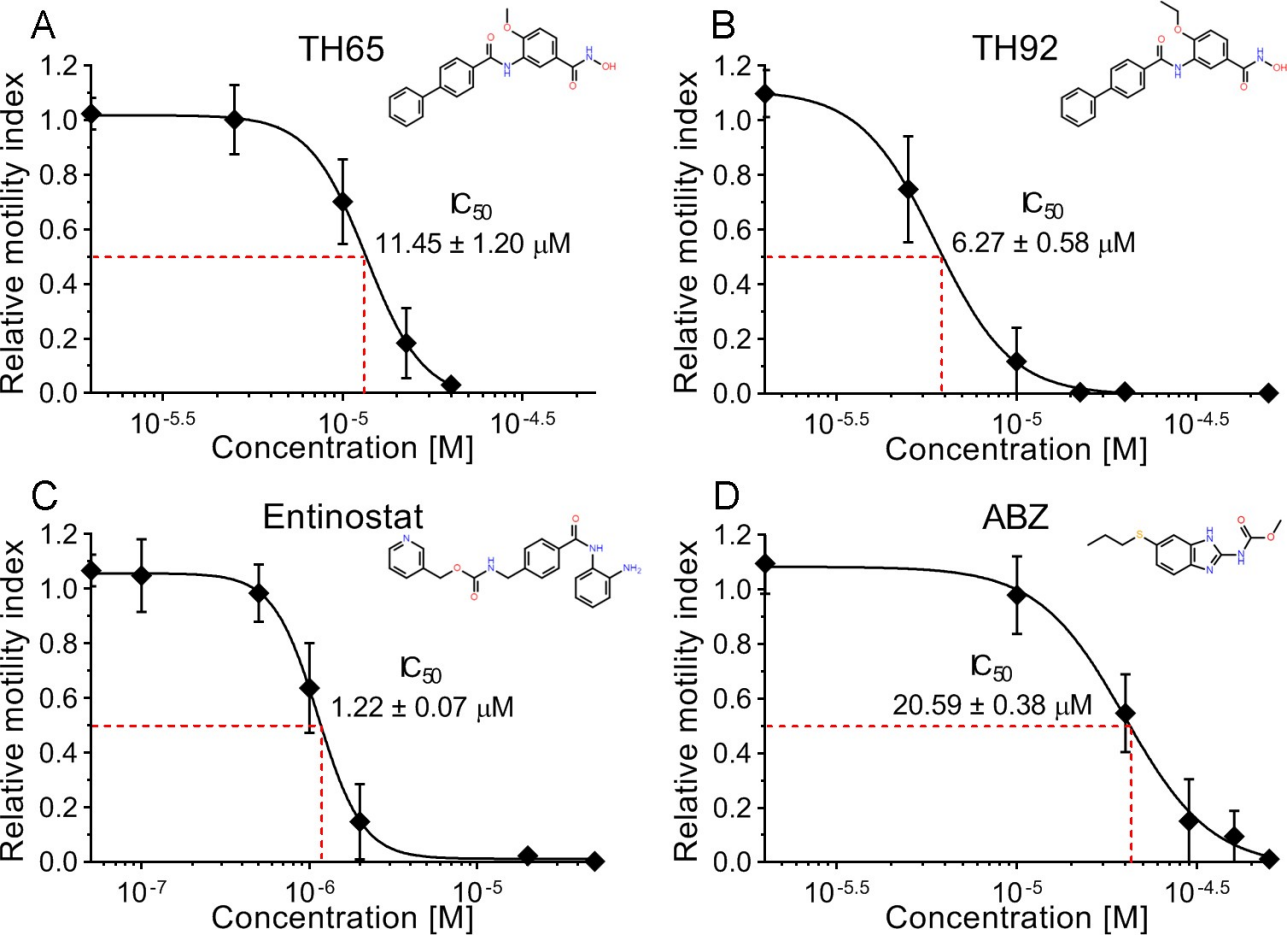
Anthelmintic dose-dependent effect of selected HDAC inhibitors and albendazole (ABZ). The anthelmintic dose-dependent effect was determined for the selected HDAC inhibitors (A) TH65, (B) TH92, and (C) entinostat, and (D) the current anthelmintic drug ABZ at 6 days of incubation from three independent biological replicates, each one in quadruplicate. Error bars represent the standard deviation. In each graph, the compounds’ chemical structure and the half-maximal inhibitory concentration (IC_50_) are shown. IC_50_ is expressed in μM, with the corresponding standard deviation.

**Fig 4:**
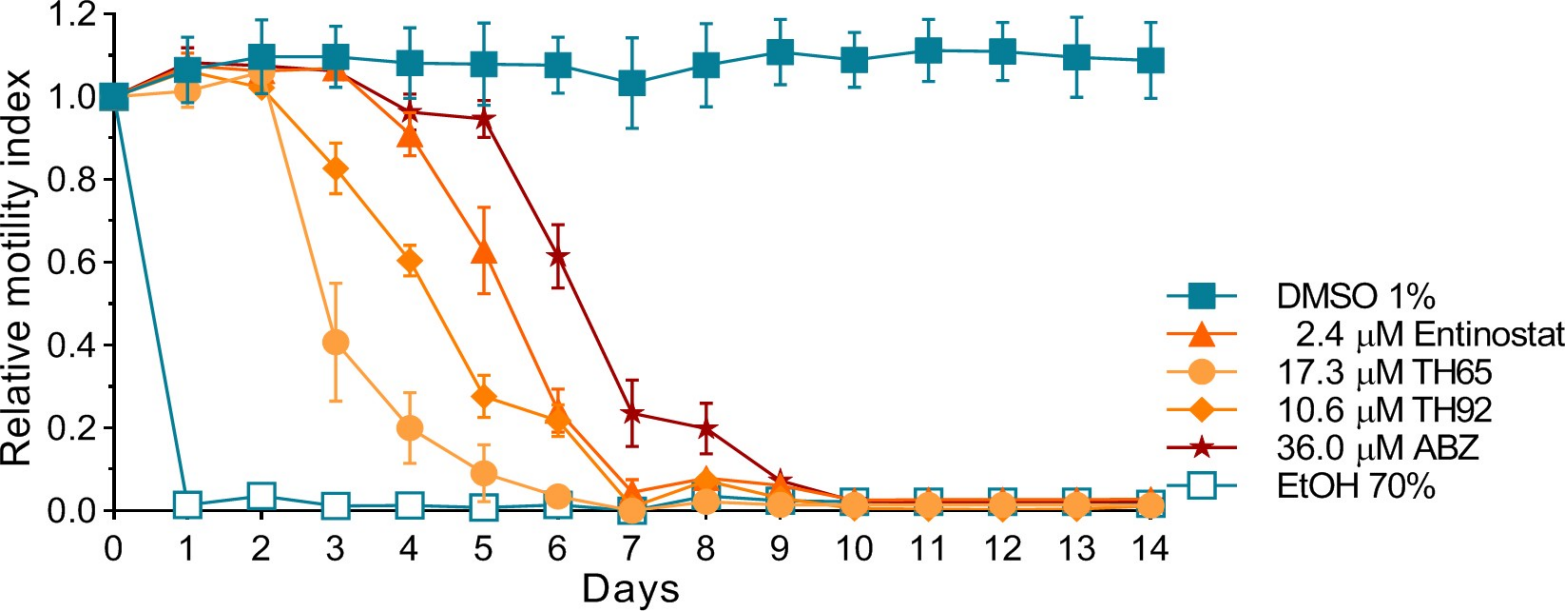
Reversibility *in vitro* test of selected HDAC inhibitors and albendazole (ABZ). *In vitro* anthelmintic activity was determined for entinostat, TH65, TH92, and ABZ at different incubation times, using the *Mesocestoides vogae* TTy (tetrathyridia) motility assay. Relative motility indices were measured from three independent biological replicates, each one in quadruplicate. Error bars represent the standard deviation. The compounds were tested at their respective IC_90_ concentrations for 6 days, then culture medium was removed, and TTy were gently washed and incubated with a fresh culture medium without adding the compounds for 8 additional days. Parasites incubated with the drug vehicle (1% DMSO) were used as a negative control.

Dose-response curves generated for entinostat, TH65, TH92, and ABZ revealed a concentration-dependent effect (Fig 3). Furthermore, the dose-response relationship parameters IC_50_, IC_25_, and IC_90_ were determined from the dose-response curves (Table 1). Each of the three HDAC inhibitors evaluated showed a potent anthelmintic effect with micromolar range activity. The individual IC_50_ values of these HDAC inhibitors were significantly lower than that of ABZ (20.58 ± 0.38 μM), being entinostat the most potent with a very low IC_50_ (1.22 ± 0.07 μM, p < 0.0001), followed by TH92 (6.27 ± 0.58 μM, p < 0.0001) and TH65 (11.45 ± 1.20 μM, p < 0.0001). Moreover, the irreversibility of the *in vitro* anthelmintic effect observed for these compounds was assessed. All evaluated compounds showed anthelmintic effects in a time-dependent manner, inducing a reduction on *M*. *vogae* TTy viability close to that expected (considering that the compounds were tested at their respective IC_90_ values), except for ABZ. No increase on parasite viability was observed after the compounds were removed from the culture medium. Moreover, the reduction of *M*. *vogae* TTy viability reached 100% before or at 10 days of incubation (Fig 4). These findings indicate that the compounds´ anthelmintic effects persisted after induction during the incubation period and were irreversible.

**Table 1:** *In vitro* anthelmintic activity of selected HDAC inhibitors and albendazole (ABZ) in *Mesocestoides vogae*.

Compound	Dose-response relationship parameters^a^		
	IC_25_ ± SD [µM]	IC_50_ ± SD [µM]	IC_90_ ± SD [µM]
Entinostat	0.91 ± 0.07	1.22 ± 0.07	2.39 ± 0.32
TH65	9.18 ± 1.84	11.45 ± 1.20	17.03 ± 3.84
TH92	4.97 ± 0.46	6.27 ± 0.58	10.58 ± 2.52
ABZ	15.82 ± 0.56	20.59 ± 0.38	36.01 ± 2.00

^a^ The reported values were calculated from three independent biological replicates, each one in quadruplicate, and were expressed with their respective standard deviations (SD).

### Selected HDAC inhibitors induced extensive and profound damage to the tegument and other structures

**Fig 5:**
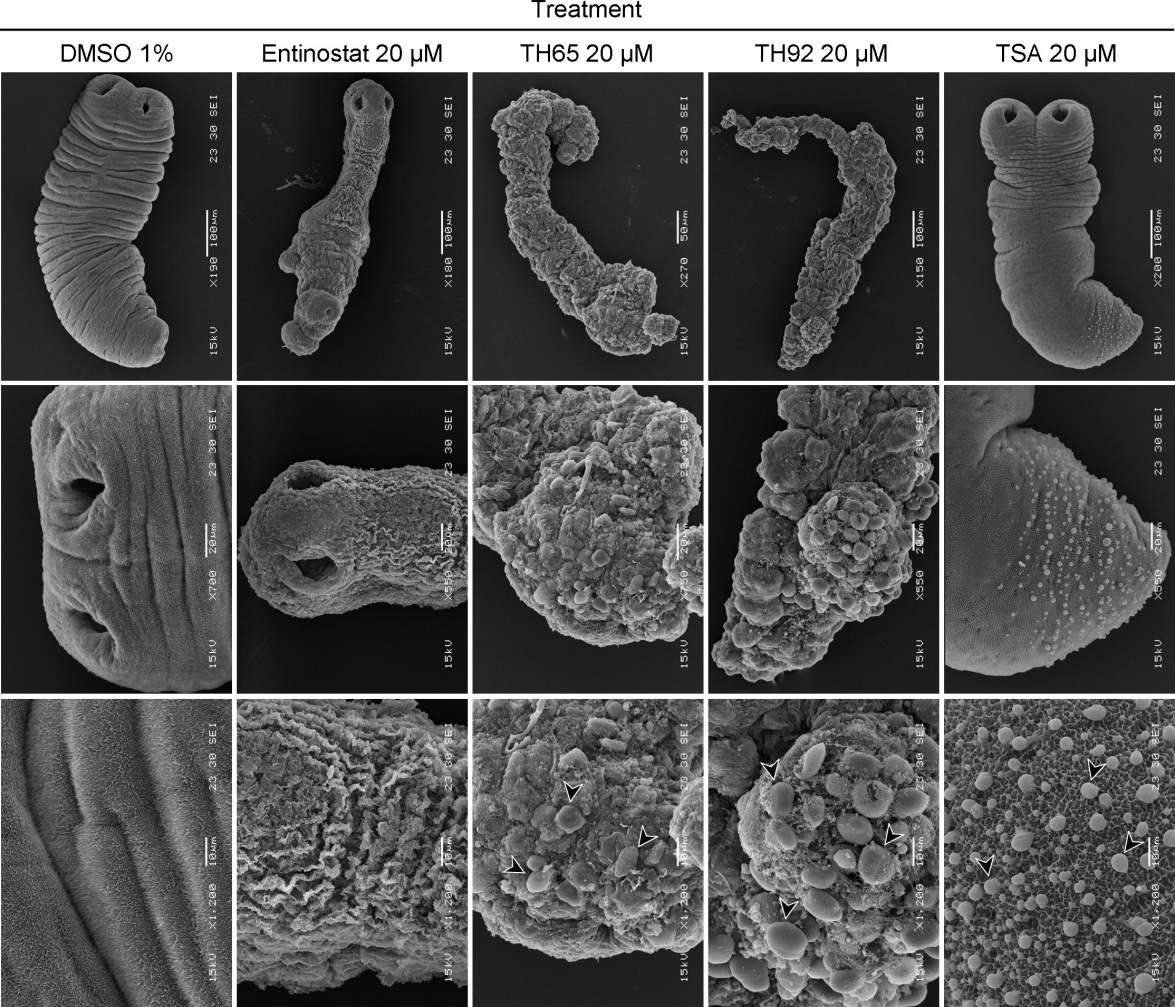
Effect in parasite morphology of selected HDAC inhibitors and trichostatin A (TSA). Scanning electron microscopy images of *Mesocestoides vogae* tetrathyridia (TTy) treated with the individual HDAC inhibitors -entinostat, TH65, and TH92- or the pan-HDAC inhibitor TSA at 20 μM after 6 days of incubation and at different magnifications (indicated in each figure). Extensive damage was observed on *M*. *vogae* TTy treated with the HDAC inhibitors, with marked alterations on the tegument and a disintegration of parasite morphology, as well as a significant number of vesicle-like structures of different sizes on the tegument (arrows) and a complete loss of microtriches. The sizes of the scale bars are shown in each image. TTy incubated with the drug vehicle 1% DMSO were used as a negative control.

SEM was used to characterize, at the ultrastructural level, changes seen in the morphology of *M*. *vogae* TTy treated with selected HDAC inhibitors or TSA (S3, S4, S5 and S6 Figs). This work showed that all selected HDAC inhibitors induced extensive damage to treated-parasites, with marked alterations on the tegument and general parasite morphology (Fig 5). *M*. *vogae* TTy incubated with TH65 and TH92 at 20 μM had more pronounced modifications, with extensive erosions on the tegument and general morphological disintegration. In these specimens, it was not possible to distinguish parasite features such as scolex, suckers, neck, and body, as previously mentioned for optical observations. Furthermore, more detailed images of the tegument of TTy treated with all evaluated HDAC inhibitors showed a large number of vesicle-like structures of different sizes and a complete loss of microtriches. These phenotypic effects were not observed in TTy incubated with the drug vehicle 1% DMSO, which were used as a negative control.

### Pairwise combinations of individual HDAC inhibitors and albendazole revealed a potent anthelmintic effect at low concentrations

**Fig 6:**
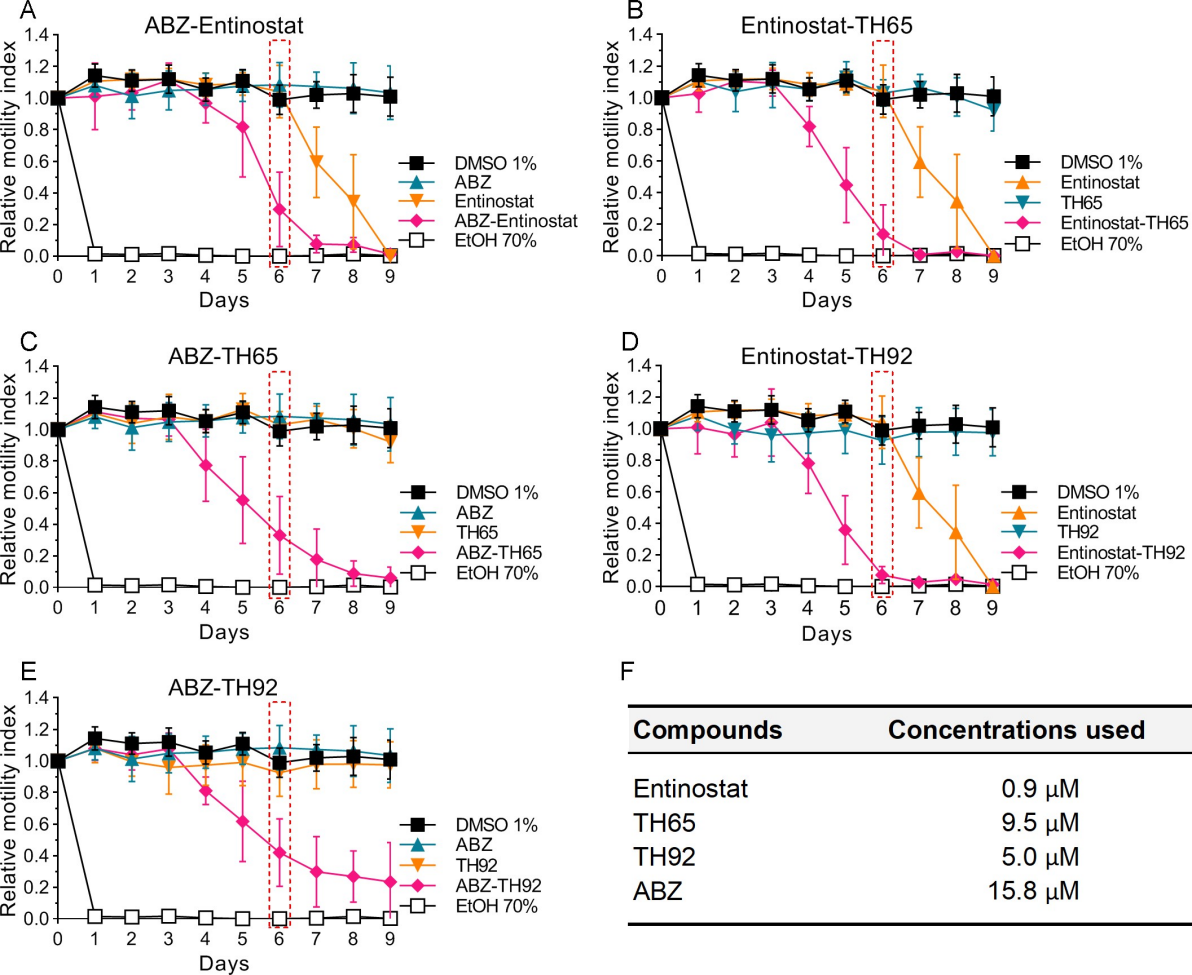
Effect of pairwise drug combinations of selected HDAC inhibitors with albendazole (ABZ) on parasite viability. *In vitro* anthelmintic activity was determined for individual HDAC inhibitors plus ABZ: (A) entinostat, (C) TH65, and (E) TH92; as well as entinostat in combination with (B) TH65, and (D) TH92. (F) Concentrations used for each compound, corresponding to their respective IC_25_ values. The compounds were tested alone and in combination at different incubation times, using the *Mesocestoides vogae* tetrathyridia (TTy) motility assay. Relative motility indices were measured from three independent biological replicates, each one in quadruplicate. Error bars represent the standard deviation. Proportional effect indices were calculated at 6 days of incubation (marked with a box). TTy incubated with the drug vehicle (1% DMSO) represented a negative control.

**Fig 7:**
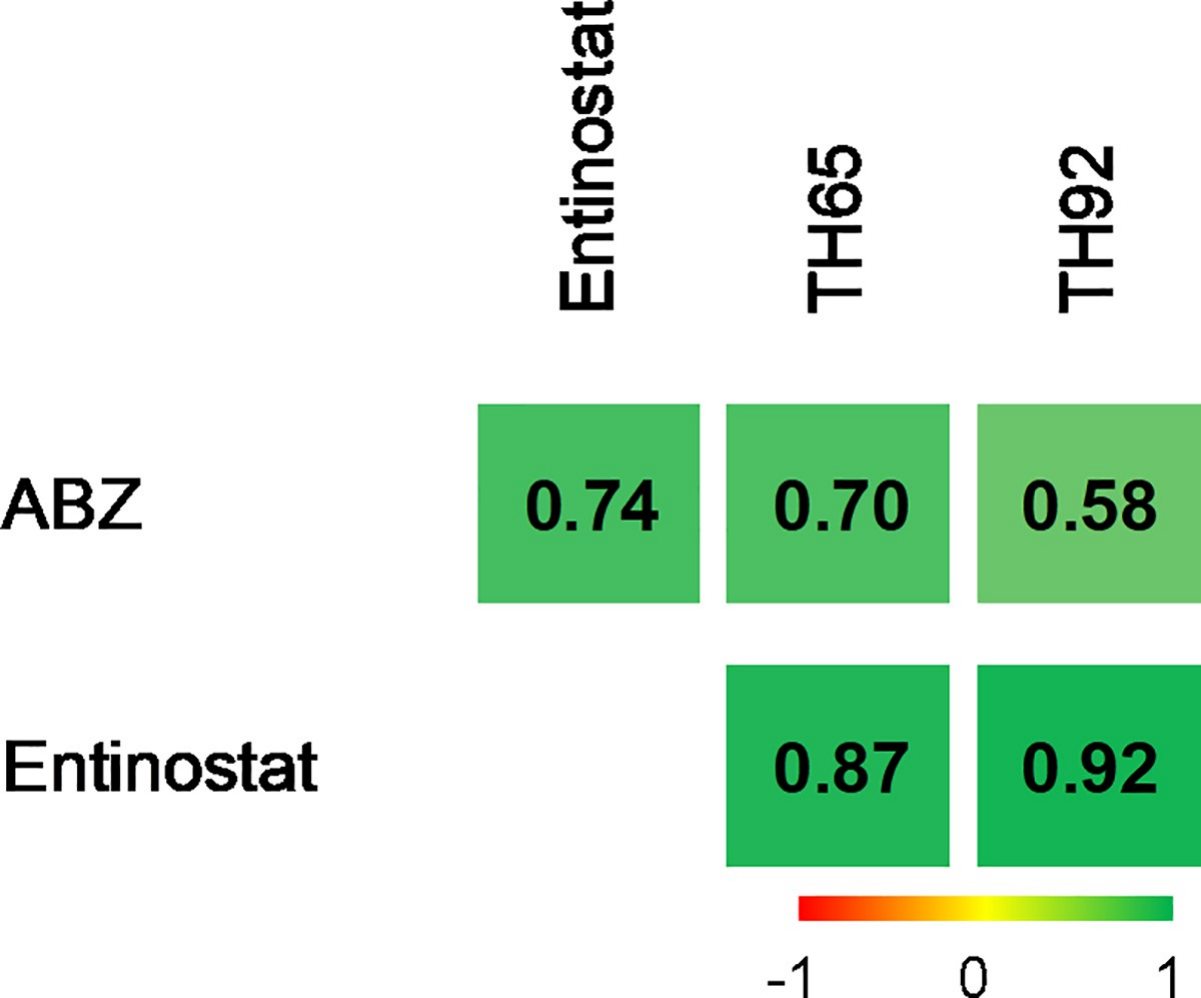
Classification of the anthelmintic effect determined for pairwise drug combinations of selected HDAC inhibitors and albendazole (ABZ). The proportional effect indices were determined for all binary drug combinations of HDAC inhibitors and ABZ tested. The values are displayed in a heat-map format to display the deviation away the different combinations’ merely additive predicted effects. Index values range from -1 to +1. The predicted additive effect index (0) is depicted in yellow. If the effect determined for the pair of compounds is higher than the predicted outcome, it is indicated in green; if the determined effect is lower, it is indicated in red. The proportional effect indices were calculated from three independent biological replicates, each in quadruplicate.

As an initial approach to evaluate whether potent HDAC inhibitors would be suitable for combination with ABZ, the *in vitro* anthelmintic effects of TH65 + ABZ, TH92 + ABZ, and entinostat + ABZ were evaluated. In addition, pairwise combinations of TH65 + entinostat and TH92 + entinostat were assessed. All pairwise combinations showed potent, time-dependent anthelmintic effects (Fig 6), while the anthelmintic effect determined for individual compounds, with the exception of entinostat, was similar to the negative control (1% DMSO). Moreover, the proportional effect indices were determined, tabulated, and displayed in a heat-map format to display the deviation away from the merely additive predicted effects. All drug combinations evaluated had high proportional effect indices (in green), as distinct from the predicted additive index (0, in yellow), suggesting positive interactions in the anthelmintic effect for individual compound pairs (Fig 7). All numerical data determined in this and previous sections of this study are shown in S1 Data.

## Discussion

Developing a highly effective and safe cestocidal compound for the treatment of cestodiases including echinococcosis and cysticercosis is critical. Recently, we identified and characterized several Class I- and II-HDAC coding genes in various cestode species and showed that the inhibition of these enzymes by the pan-HDAC inhibitor TSA affected *M*. *vogae* TTy, leading to decreased viability and significant morphological alterations, as well as increased acetylation of total proteins (including histone H4); these findings suggested that HDACs could represent drug targets in cestodes [27].

In the present study, we determined and characterized the *in vitro* effects of a panel of compounds against Class I- and Class II-HDAC enzymes using the *M*. *vogae* TTy motility assay, and described optical microscopic observations. Phenotypic screening showed that several of these compounds displayed a significant anthelmintic effect in a time- and dose-dependent manner, resulting in a decrease on parasite viability and extensive damage on the tegument, with loss of parasite integrity. Some of the Class I-HDAC inhibitors evaluated led to the death of TTy before 9 days of treatment at several concentrations, suggesting that these compounds represent new candidate drugs against cestodes. Additionally, there was a correlation between the intensity of morphological damage and TTy viability. The inhibitors analyzed in this study likely affect *M*. *vogae* viability via HDAC inhibition, as previously observed for TSA [27]. However, this proposal remains to be tested. Furthermore, various series of structure-based designed inhibitors against SmHDAC8 [30–32] were also evaluated. Several benzhydroxamate derivatives (series TH) showed a potent anthelmintic effect. Compounds TH65 and TH92 each induced severe damage to the tegument and morphological disintegration. These compounds have shown some selective activity on SmHDAC8 over the major *H*. *sapiens* HDAC isoforms (HDAC1 and HDAC6), although the activities against SmHDAC8 and the human HDAC8 were similar [30]. HDACs inhibitors showing a preference for one isoform have been proposed as therapeutic compounds in cancer and other diseases, including parasitic diseases [30, 43–46]. This is because isoform-selective inhibitors can alter distinct pathways more specifically involved in disease mechanisms, contrary to broad-spectrum HDAC inhibitors, which can alter multiple cellular processes, thus causing higher toxic effects [43]. In addition, the compounds showed relatively low cytotoxicity levels in cellular assays [30]. For TH65, the IC_50_ for the toxicity in HEK293 cells was 199 μM, and for TH92, this value was > 300 μM, indicating that both compounds do not show overt toxicity against this human cell line. Although it will be necessary to achieve high parasite selectivity to prepare the compounds for clinical use, the results of the present study are promising and pave the way for a rational design of selective drugs against parasite HDAC8 to treat NTDs caused by cestodes. In agreement with our results, a decrease on parasite viability was observed after incubating the juvenile forms of *S*. *mansoni* with TH65 and TH92 [30]. The anthelmintic effects determined here were comparable in concentration and incubation time to that determined for TH65 on *S*. *mansoni* viability (by the AlamarBlue assay). TH92 was more potent on *M*. *vogae* TTy than on *S*. *mansoni* juvenile forms [30]. This difference could be due to a higher affinity of TH92 for HDAC8 from *M*. *vogae* than SmHDAC8, or a higher capacity for intra-parasitic diffusion/accumulation of this compound on *M*. *vogae* TTy. The other compounds of the series TH were less potent against *M*. *vogae* TTy. The anthelmintic effects determined here for some of them were not comparable in concentration and/or incubation time to those determined on *S*. *mansoni* viability. This could be due to differences in the affinity of these compounds for HDAC8 from *M*. *vogae* compared to SmHDAC8, showing the importance of synthesizing and evaluating various derivatives containing different substituents for each structural compound series. Furthermore, various Class II-HDAC inhibitors were also evaluated. The compounds JS16, JS28, KK16, and ST36 did not show a significant effect on *M*. *vogae* TTy viability at any of the tested concentrations, even up to 9 days of treatment. These compounds have been designed as HDAC inhibitors to the HDAC6 from *H*. *sapiens* (HsHDAC6), showing a selective activity for HsHDAC6 compared with other human HDAC isoforms. Additionally, cytotoxicity studies in several human cell lines have shown a significant effect on cell proliferation for these compounds [47–49]. The null effect on *M*. *vogae* TTy viability determined here for these compounds could be due to differences in the affinity for HDAC6 from *M*. *vogae* compared with HsHDAC6. The HDAC6 genes are transcribed in several development stages in *Echinococcus* spp. There is limited sequence identity (< 51%) in the HDAC catalytic domains between cestode HDAC6s and HsHDAC6 [27]. For these reasons, a better characterization of HDAC6s would be important in working toward developing new cestocidals. Finally, the anthelmintic drugs PZQ and ABZ, and the pan-HDAC inhibitor TSA, were also evaluated. The results obtained for these compounds were similar to those reported previously [27], which allowed us to set up *M*. *vogae* TTy viability determination methods, and use these compounds as control drugs.

Due to the limited availability of biological material of zoonotic cestodes, such as *Echinococcus granulosus* and *Taenia solium*, we used TTy of *M*. *vogae* as a model to determine the potential cestocidal properties of various compounds. *M*. *vogae* TTy have a remarkable capacity for asexual reproduction in the peritoneal cavity of mice, providing continuous biological material availability. It is also easily cultured and is regarded as non-infective for humans [50, 51]. This model has been used to identify new cestocidal compounds in pharmacological studies [39, 52–56]. Using *M*. *vogae* TTy to screen HDAC inhibitors as cestocidal compounds allowed us to select candidates to be later tested in experimental animal models of the zoonotic parasites, such as *E*. *granulosus*. This is a necessary step in developing new chemotherapeutic alternatives since the efficacy of the compounds may differ among cestodes belonging to different families. Another challenge is the localization of the larval stages of taeniid cestodes in the intermediate host. For example, *E*. *granulosus* cysts localize mainly in the liver and lungs, and *T*. *solium* cysticerci in the central nervous system, which is distinct from adult worms which dwell in the small intestines of definitive hosts. Taking this into account, murine models of hepatic echinococcosis [57] and cerebral/cerebellum neurocysticercosis [58, 59] should be used in the future.

In addition to investigating the cestocidal profile of 20 Class I- and Class II-HDAC inhibitors against TTy *in vitro*, we also characterized the anthelmintic effect of the most potent HDAC inhibitors on *M*. *vogae* using three different approaches: evaluation of the anthelmintic dose-response *in vitro* effect (Fig 3 and Table 1), evaluation of the reversibility (Fig 4) and evaluation of the impact on worm morphology at an ultrastructural level (Fig 5). Entinostat, TH65, and TH92 were selected because these compounds displayed potent activity against *M*. *vogae* TTy. Selected HDAC inhibitors showed a potent effect in a concentration-dependent manner with activities in the micromolar range. Interestingly, the IC_50_ values determined for these compounds were significantly lower than those determined for the approved anthelmintic drug ABZ. Similar to our results, TH65 and TH92 provoked a marked reduction of *S*. *mansoni* schistosomula viability, with an IC_50_ value for the first compound in the micromolar range [30]. Entinostat had the most potent effect with IC_50_ being lower than for ABZ. Since this compound is undergoing phase III clinical trials to treat various cancers and has been granted breakthrough therapy status by the FDA [60], entinostat deserves further consideration as a cestocidal drug. However, taking into account that in clinical trials the maximum plasma concentration of entinostat was 53 ng/ml (0.14 μM), when the maximum tolerated dose was used [61], it will likely be necessary to adjust the dose regime and/or try combinations of entinostat with currently used anthelmintic drugs, such as ABZ, to determine whether the desired efficacy using maximum tolerated levels of entinostat can be achieved.

SEM was used to characterize changes in the morphology and ultrastructural features on *M*. *vogae* TTy treated with selected HDAC inhibitors or TSA. The main difference observed after exposure to TH65, TH92 or entinostat was extensive damage on the tegument of *M*. *vogae* TTy and their morphological disintegration. The alterations were consistent with death and were more pronounced for TH65 and TH92 compared with entinostat. Furthermore, a large number of vesicle-like structures were observed on the tegument of TTy treated with each of the HDAC inhibitors evaluated, as well as a complete loss of microtriches. Microtriche destruction would likely interfere with nutritional uptake into TTy, as these structures are directly associated with the active absorption of nutrients. These phenotypic alterations were similar to those observed on *M*. *vogae* TTy treated with PZQ [50] or thymol [39, 62].

Finally, we studied the pharmacological potential of pairwise combinations of the HDAC inhibitors TH65, TH92 and entinostat with ABZ; as well as the combinations of TH65 and TH92 with entinostat. All drug combinations showed potent anthelmintic effects in a time-dependent manner. In general, drugs with synergic activity can act on different sites of the same target molecule, on different targets of the same pathway, or on different targets belonging to one or more connected pathways. In the present study, positive interactions were observed for the combinations of ABZ with the selected HDAC inhibitors. Although we do not know the mechanisms allowing the positive interaction for these two types of compounds tested on *M*. *vogae*, in other diseases such as cancer, synergic effects of combination of microtubule-destabilizing agents and HDAC inhibitors have been described [63, 64]. Those combinations caused cell cycle arrest followed by apoptosis, since microtubule dynamics as well as HDAC activity are involved in cell cycle progression. With respect to the combination of different HDAC inhibitors, even though they seem to have a similar mechanism of action, they could inhibit different HDAC enzymes involved in the same or related pathways. It is known that entinostat is highly selective for HDAC1 (in the nanomolar range), but significantly less so for HDAC2 and HDAC3 (micromolar range). Additionally, no activity against HDAC8 or any Class II-HDAC enzymes was reported for this compound [65, 66]. In contrast, TH65 and TH92, are highly selective for HDAC8 over HDAC1 and HDAC6 [30]. The different phenotypes observed could suggest different mechanisms of action for each of the compounds tested, which, in combination, would ‘positively’ interact, rather than act in an additive way. However, it is important to mention that these results do not necessarily mean that the observed, positive interactions reached the level of being “synergistic”. Synergistic effect, if confirmed, could reduce a compound´s concentration needed for treatment and reduce potential secondary side effects caused by most of the anthelmintic drugs used. The method used here was adapted from a simplified protocol by Planer et al. [40]. In this study [40], 8 of the most promising 23 apparent synergistic combinations were shown to relate to true synergism by isobologram studies. Another limitation found was that, although all combination showed potent anthelmintic *in vitro* effects, no effect on parasite viability was observed for individual compounds at their calculated IC_25_ values, with the exception of entinostat. This result was confirmed by visual inspection, and could be attributed to differences in the batches of samples used. Different batches of worms could display differing sensitivity to low drug concentrations due to their heterogeneous nature. For all of these reasons, while the initial findings are promising, they should be considered with caution and confirmed further in isobologram studies.

In conclusion, this first report of the anthelmintic profile of HDAC inhibitors against *M*. *vogae* TTy showed that several of these inhibitors have a potent anthelmintic *in vitro* activity, produce a significant reduction on *M*. *vogae* TTy viability and/or induce major alterations on the tegument and/or other morphological structures. These results have allowed us to select promising compounds (TH65, TH92 and entinostat) as candidates for further evaluation and optimization. These compounds demonstrated a dose-dependent, potent and irreversible anthelmintic effect. The compounds tested also showed potencies that were higher than ABZ. Finally, testing each of these selected HDAC inhibitors combined with ABZ showed potent anthelmintic effects in a time-dependent manner at low concentrations (0.9–5.0 μM). The results obtained here provide a foundation for optimization *via* structure-activity relationship (SAR) studies, and *in vivo* efficacy and toxicity studies in experimentally infected animals. The evaluation and characterization of various inhibitors against HDAC enzymes from different classes should contribute to an understanding the role of these enzymes in cestodes and thus, aid in the development of new treatments against human cestodiases, including echinococcosis and cysticercosis.

## Supporting information

S1 Table List of histone deacetylase (HDAC) inhibitors evaluated in this work. (XLSX)

S1 Text Supplementary methods. (DOCX)

S1 Data Excel spreadsheet containing, in separate sheets, the underlying numerical data for Figs 1A, 1B, 1C, 1D, 1E, 1F, 1G, 1H, 2A, 2B, 2C, 2D, 2E, 2F, 2G, 2H, 2I, 2J, 2K, 2L, 3A, 3B, 3C, 3D, 4, 6A, 6B, 6C, 6D, 6E, S1 and S2. (XLSX)

S1 Fig Effect of the drug vehicle dimethylsulfoxide (DMSO) on the viability of *Mesocestoides vogae* tetrathyridia (TTy). Effect of DMSO at 1% on parasite viability at different incubation times in comparison to culture medium without DMSO, determined by the *M*. *vogae* TTy motility assay. Relative motility indices were measured from three independent biological replicates, each one in quadruplicate. Error bars represent the standard deviation and the asterisks indicate those values that showed differences with statistical significance compared to the negative control without DMSO, according to two-way ANOVA test.
(TIF)

S2 Fig Controls used in the anthelmintic effect evaluation of HDAC inhibitors on *Mesocestoides vogae* tetrathyridia (TTy). *In vitro* anthelmintic activity was determined for the current anthelmintic drugs praziquantel (PZQ) and albendazole (ABZ) at 20 μM, the pan-HDAC inhibitor trichostatin A (TSA) at 20 μM, the drug vehicle dimethylsulfoxide (DMSO) 1% and ethanol (EtOH) 70% at different incubation times, using the *M*. *vogae* TTy motility assay. These compounds were used as control in the anthelmintic effect determination of HDAC inhibitors. Relative motility indices were measured from three independent biological replicates, each one in quadruplicate. Error bars represent the standard deviation and the asterisks indicate those values that showed differences with statistical significance compared to the negative control (DMSO 1%), according to two-way ANOVA test and Dunnett’s post-tests (*, p < 0.05; **, p < 0.01; ***, p < 0.001; ****, p < 0.0001).
(TIF)

S3 Fig Effect of the compounds used as control drugs on the morphology of *Mesocestoides vogae* tetrathyridia (TTy). Inverted optical microscope images of *M*. *vogae* TTy treated with the current anthelmintic drugs, praziquantel (PZQ) and albendazole (ABZ), and the pan-HDAC inhibitor trichostatin A (TSA) at 20 μM at different days of treatment; compared to the parasites incubated with DMSO 1%. Note the extensive damage on the tegument with the presence of blebs (arrows) and loss of general parasite morphology, as well as the presence of influx (I) of culture medium into the worm and tegument debris (R) in the culture medium. These phenotypic alterations were observed for three independent biological replicates and were marked in the images at 9 days of treatment. Scale bars represent 100 μm.
(TIF)

S4 Fig Effect of the Class I-HDAC inhibitor entinostat on the morphology of *Mesocestoides vogae* tetrathyridia (TTy). Inverted optical microscope images of *M*. *vogae* TTy treated with entinostat at 2, 20, and 50 μM and at different days of treatment; compared to the parasites incubated with DMSO 1%. Note the extensive damage on the tegument with the presence of blebs (arrows) and loss of general parasite morphology, as well as the presence of some constrictions on the body (circles), influx (I) of culture medium into the worm and tegument debris (R) in the culture medium. These phenotypic alterations were observed for three independent biological replicates and were marked in the images at 9 days of treatment. Scale bars represent 100 μm.
(TIF)

S5 Fig Effect of the Class I-HDAC inhibitor TH65 on the morphology of *Mesocestoides vogae* tetrathyridia (TTy). Inverted optical microscope images of *M*. *vogae* TTy treated with TH65 at 2, 20, and 50 μM and at different days of treatment; compared to the parasites incubated with DMSO 1%. Note the extensive damage on the tegument with the presence of blebs (arrows) and the complete loss of general parasite morphology, as well as the presence of the influx (I) of culture medium into the worm and tegument debris (R) in the culture medium. These phenotypic alterations were observed for three independent biological replicates and were marked in the images at 9 days of treatment. Additionally, note the formation of crystal-like structures in the culture medium whit TH65 at 50 μM after 1 day of treatment (marked with circles). Scale bars represent 100 μm.
(TIF)

S6 Fig Effect of the Class I-HDAC inhibitor TH92 on the morphology of *Mesocestoides vogae* tetrathyridia (TTy). Inverted optical microscope images of *M*. *vogae* TTy treated with TH92 at 2, 20, and 50 μM and at different days of treatment; compared to the parasites incubated with DMSO 1%. Note the extensive damage on the tegument with the presence of blebs (arrows) and the complete loss of general parasite morphology, as well as the presence of the influx (I) of culture medium into the worm and tegument debris (R) in the culture medium. These phenotypic alterations were observed for three independent biological replicates and were marked in the images at 9 days of treatment. Scale bars represent 100 μm.
(TIF)
