# Supplementary material for: The potential for histone deacetylase (HDAC) inhibitors as cestocidal drugs
Source: PLoS Negl Trop Dis. 2021 Mar 3;15(3):e0009226. doi: 10.1371/journal.pntd.0009226 (PMC7959350; doi:10.1371/journal.pntd.0009226)
Supplement: S1 Text — (DOCX) [file pntd.0009226.s002.docx]

# Experimental methods

Synthesis of HDAC inhibitors

1. General

All materials and reagents were purchased from Sigma-Aldrich Co. Ltd. and ABCR GmbH. All solvents were analytically pure and dried before use. Thin layer chromatography was carried out on aluminum sheets coated with silica gel 60 F254 (Merck, Darmstadt, Germany). For column chromatography under normal pressure silica gel 60 (0.036−0.200 mm) was used.

Purity of the final compounds was assessed by HPLC, and was measured by UV absorbance at 254 nm. The HPLC consists of an XTerra RP18 column (3.5 μm, 3.9 mm × 100 mm) from the manufacturer Waters (Milford, MA, USA) and two LC-10AD pumps, a SPD-M10A VP PDA detector, and a SIL-HT autosampler, all from the manufacturer Shimadzu (Kyoto, Japan).

Mass spectrometry analyses were performed with a Finnigan MAT710C (Thermo Separation Products, San Jose, CA, USA) for the ESIMS spectra and with a LTQ (linear ion trap) Orbitrap XL hybrid mass spectrometer (Thermo Fisher Scientific, Bremen, Germany) for the HRMS-ESI (high resolution mass spectrometry) spectra. For the HRMS analyses the signal for the isotopes with the highest prevalence was given and calculated (^35^Cl, ^79^Br).

^1^H NMR and ^13^C NMR spectra were taken on a Varian Inova 400 using deuterated DMSO as solvent. Chemical shifts are referenced to the residual solvent signals. The following abbreviations and formulas for solvents and reagents were used: ethyl acetate (EtOAc), dimethylformamide (DMF), methanol (MeOH), tetrahydrofuran (THF), chloroform (CHCl_3_), dichloromethane (CH_2_Cl_2_), N,N-diisopropylethylamine (DIPEA), trimethylamine (TEA), hydrochloric acid (HCl), Benzotriazol-1-yl-oxytripyrrolidinophosphonium-hexafluorophosphat (PyBOP) and trifluoroacetic acid (TFA).

In vitro testing of all final inhibitors was carried out on recombinant human HDAC1, HDAC6, HDAC8 as well as *Schistosoma mansoni* HDAC8. Results have been published elsewhere [1,2].

2. Scheme for the synthesis of the final compounds

3. Synthesis of the intermediates

TH119a and TH138a were commercially available. EG13b, EG18a, EG20a, TH138b and TH139b were prepared as previously described [1,2]. EG13a was synthesized according to the method described by Unzue [3] with the modification of using n-propyl iodide as alkylating agent.

EG18b

3-Amino-4-ethoxyacetophenon

The title compound was prepared through the nitration of 4-hydroxyacetophenone as previously described [4]. The nitro derivative was then alkylated with iodoethane following the protocol reported in [3]. Finally 4-ethoxy-3-nitroacetophenon was reduced using stannous chloride to afford 3-Amino-4-ethoxyacetophenon.

TH119b

Methyl 3-amino-4-(methylthio)benzoate

It was prepared from 4-(methylthio)-3-nitrobenzoic acid -using thionyl chloride- according to the same method as for the other methyl esters [1,2]. Methyl 4-(methylthio)-3-nitrobenzoate was then reduced using stannous chloride to afford the title compound.

4. Synthesis of carboxylic acids (CA) precursors of the hydroxamic acids

The appropriate carboxylic acid (1 eq.), DMF (one drop) and oxalyl chloride (1.3 eq.) were stirred in CH_2_Cl_2_ at room temperature for 3 h. The mixture was then added dropwise to a solution of the appropriate amine (0.9 eq.) and DIPEA (3 eq.) in CH_2_Cl_2_, and stirring was continued for another 2 h at room temperature. The reaction mixture was washed with a saturated aqueous solution of ammonium chloride and brine. The organic layer was dried over anhydrous sodium sulfate, filtered, and concentrated in vacuo. The residue was purified using column chromatography (CHCl_3_: MeOH, 100:0-98:2).

The methyl ester product (1 eq.) was dissolved in MeOH followed by the addition of aqueous solution of sodium carbonate (10 eq.) and the mixture was stirred under reflux for 2-4 h until complete hydrolysis of the ester. The solvents were then evaporated and the reaction was neutralized with aqueous solution of 1M HCl until pH 6. The liberated free acid was extracted with a mixture of ethyl acetate and THF, and the extract was dried over sodium sulfate and evaporated under reduced pressure. The product required no further purification.

5. Synthesis of hydroxamic acids

The appropriate carboxylic acid (1 eq.) was dissolved in dry THF, followed by the addition of PyBOP (1.2 eq.) and DIPEA (3 eq.). The mixture was stirred for 15 min, then NH_2_OTHP (1.2 eq.) was added and the reaction mixture was stirred at room temperature for 2-4 h. The solvent was evaporated under vacuum and the mixture was dissolved in EtOAc (50 ml) and washed with 1M sodium carbonate solution and brine. The organic layer was evaporated under vacuum and the product was purified by column chromatography (CHCl_3_: MeOH: formic acid, 99:0.95:0.05).

The obtained product was dissolved in THF and a catalytic amount of conc. HCl was added and the reaction was controlled by TLC. After that the solvent was evaporated under vacuum and the hydroxamic acid product was purified by column chromatography (CHCl_3_: MeOH, TEA 95:4.95:0.05).

**Full analytical characterization of the final compounds:**

**EG13**

3-(5-acetyl-2-propoxybenzamido)-4-methylbenzohydroxamic acid

MS m/z: 369.47 [M-H]^-^

^1^H NMR (400 MHz, DMSO) δ 11.16 (s, 1H), 9.76 (s, 1H), 8.96 (s, 1H), 8.31 (d, *J* = 2.3 Hz, 1H), 8.16 – 8.01 (m, 2H), 7.49 (dd, *J* = 7.9, 1.5 Hz, 1H), 7.32 (d, *J* = 8.6 Hz, 2H), 4.22 (t, *J* = 6.6 Hz, 2H), 2.57 (s, 3H), 2.31 (s, 3H), 1.92 – 1.74 (m, 2H), 0.97 (t, *J* = 7.4 Hz, 3H).

HRMS m/z: 371.1610 [M+H]^+^; calculated C_20_H_23_N_2_O_5_^+^: 371.1606

HPLC: rt 10.18 min (purity 99.26 %)

Yield: 230 mg; 0.62 mmol; 18 %

**EG18**

3-(5-acetyl-2-ethoxyphenylcarbamoyl)-4-chlorobenzhydroxamic acid

MS m/z: 375.35 [M-H]^-^

^1^H NMR (400 MHz, DMSO) δ 11.38 (s, 1H), 9.84 (s, 1H), 9.16 (s, 1H), 8.44 (d, *J* = 1.7 Hz, 1H), 7.98 (s, 1H), 7.91 – 7.78 (m, 2H), 7.64 (d, *J* = 8.3 Hz, 1H), 7.17 (d, *J* = 8.6 Hz, 1H), 4.18 (q, *J* = 6.9 Hz, 2H), 2.53 (s, 3H), 1.36 (t, *J* = 7.0 Hz, 3H).

HRMS m/z: 377.0892 [M+H]^+^; calculated C_18_H_18_ClN_2_O_5_^+^: 377.0904

HPLC: rt 9.44 min (purity 99.07 %)

Yield: 100 mg; 0.26 mmol; 17 %

**EG20**

3-(5-acetyl-2-ethoxyphenylcarbamoyl)-4-methylbenzhydroxamic acid

MS m/z: 355.45 [M-H]^-^

^1^H NMR (400 MHz, DMSO) δ 11.23 (s, 1H), 9.55 (s, 1H), 9.03 (s, 1H), 8.34 (s, 1H), 7.91 (s, 1H), 7.83 (dd, *J* = 8.6, 2.2 Hz, 1H), 7.75 (dd, *J* = 7.9, 1.7 Hz, 1H), 7.37 (d, *J* = 8.0 Hz, 1H), 7.17 (d, *J* = 8.7 Hz, 1H), 4.18 (q, *J* = 6.9 Hz, 2H), 2.53 (s, 3H), 2.46 (s, 3H), 1.36 (t, *J* = 7.0 Hz, 3H).

HRMS m/z: 357.1442 [M+H]^+^; calculated C_19_H_21_N_2_O_5_^+^: 357.1450

HPLC: rt 8.77 min (purity 99.84 %)

Yield: 340 mg; 0.95 mmol; 36 %

**TH119**

3-(4-Biphenylamido)-4-methylthiobenzhydroxamic acid

MS m/z: 377.31 [M-H]^-^

^1^H NMR (400 MHz, dmso) δ 11.24 (s, 1H), 10.04 (s, 1H), 9.02 (s, 1H), 8.11 – 8.02 (m, 2H), 7.88 – 7.80 (m, 2H), 7.79 – 7.73 (m, 3H), 7.73 – 7.67 (m, 1H), 7.54 – 7.46 (m, 2H), 7.45 – 7.37 (m, 2H), 3.34 (s, 3H).

HRMS m/z: 401.0921 [M+Na]^+^; calculated C_21_H_18_N_2_O_3_SNa^+^ 401.0930

HPLC: rt 9.42 min (purity 91.17%)

Yield: 90 mg; 0.24 mmol; 20 %

**TH138**

3-[2-Chlor-4-(4-fluorphenyl-)-benzamido]-4-methoxybenzhydroxamic acid

MS m/z: 413.25 [M-H]^-^

^1^H NMR (400 MHz, dmso) δ 11.12 (s, 1H), 9.76 (s, 1H), 8.91 (s, 1H), 8.38 (s, 1H), 7.89 – 7.55 (m, 6H), 7.32 (t, J = 8.6 Hz, 2H), 7.12 (d, J = 8.4 Hz, 1H), 3.86 (s, 3H).

HRMS m/z: 437.0666 [M+Na]^+^; calculated C_21_H_16_FClN_2_O_4_Na^+^ 437.0675

HPLC: rt 11.81 min (purity 98.44%)

Yield: 75 mg; 0.18 mmol; 15 %

**TH139**

3-[2-Chlor-4-(4-fluorphenyl-)-benzamido]-4-chlorbenzhydroxamic acid

MS m/z: 417.17 [M-H]^-^

^1^H NMR (400 MHz, dmso) δ 11.39 (s, 1H), 10.37 (s, 1H), 9.15 (s, 1H), 8.12 (s, 1H), 7.94 – 7.57 (m, 7H), 7.41 – 7.22 (m, 2H).

HRMS m/z: 441.0173 [M+Na]^+^; calculated C_20_H_13_FCl_2_N_2_O_3_Na^+^ 441.0180

HPLC: rt 11.81 min (purity 94.00%)

Yield: 60 mg; 0.14 mmol; 18 %

References:

1. Heimburg T, Chakrabarti A, Lancelot J, Marek M, Melesina J, Hauser A-T, et al. Structure-Based Design and Synthesis of Novel Inhibitors Targeting HDAC8 from *Schistosoma mansoni* for the Treatment of Schistosomiasis. J Med Chem. 2016;59: 2423–2435. doi:10.1021/acs.jmedchem.5b01478

2. Heimburg T, Kolbinger FR, Zeyen P, Ghazy E, Herp D, Schmidtkunz K, et al. Structure-Based Design and Biological Characterization of Selective Histone Deacetylase 8 (HDAC8) Inhibitors with Anti-Neuroblastoma Activity. J Med Chem. 2017;60: 10188–10204. doi:10.1021/acs.jmedchem.7b01447

3. Unzue A, Zhao H, Lolli G, Dong J, Zhu J, Zechner M, et al. The ‘gatekeeper’ Residue Influences the Mode of Binding of Acetyl Indoles to Bromodomains. J Med Chem. 2016;59: 3087–3097. doi:10.1021/acs.jmedchem.5b01757

4. Wang S, Yan J, Wang J, Chen J, Zhang T, Zhao Y, et al. Synthesis of some 5-phenylisoxazole-3-carboxylic acid derivatives as potent xanthine oxidase inhibitors. Eur J Med Chem. 2010;45: 2663–2670. doi:10.1016/j.ejmech.2010.02.013
