# Supplementary figures and images for: The potential for histone deacetylase (HDAC) inhibitors as cestocidal drugs
Source: PLoS Negl Trop Dis. 2021 Mar 3;15(3):e0009226. doi: 10.1371/journal.pntd.0009226 (PMC7959350; doi:10.1371/journal.pntd.0009226)

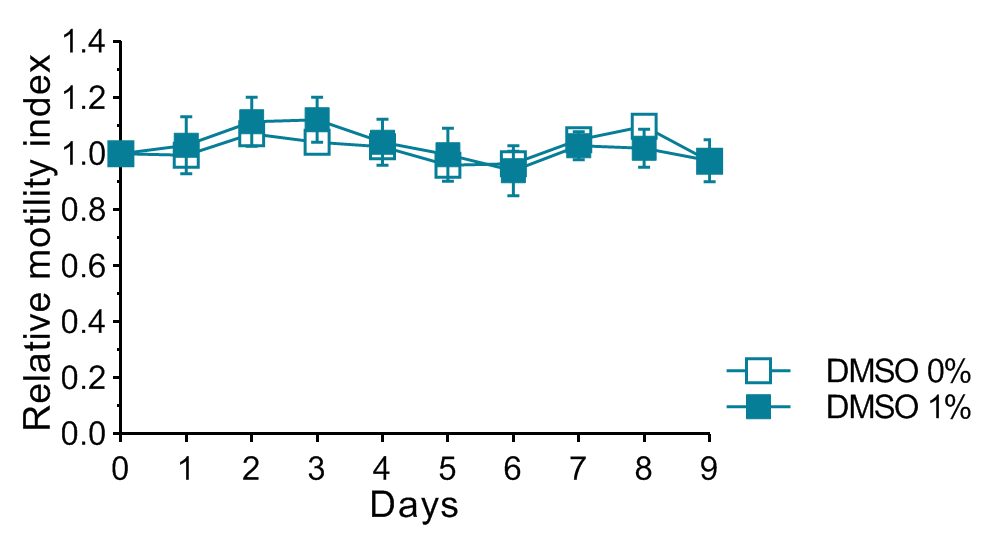

Supplement: S1 Fig — Effect of DMSO at 1% on parasite viability at different incubation times in comparison to culture medium without DMSO, determined by the M. vogae TTy motility assay. Relative motility indices were measured from three independent biological replicates, each one in quadruplicate. Error bars represent the standard deviation and the asterisks indicate those values that showed differences with statistical significance compared to the negative control without DMSO, according to two-way ANOVA test. (TIF) [file pntd.0009226.s004.tif]

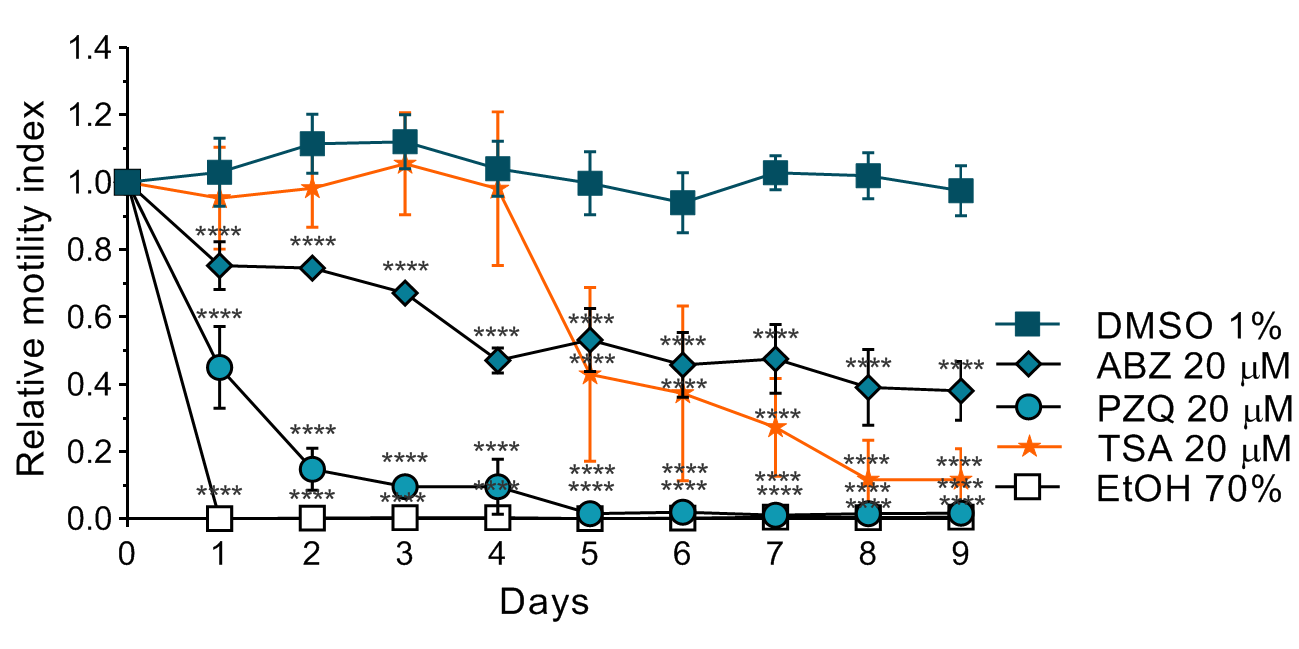

Supplement: S2 Fig — In vitro anthelmintic activity was determined for the current anthelmintic drugs praziquantel (PZQ) and albendazole (ABZ) at 20 μM, the pan-HDAC inhibitor trichostatin A (TSA) at 20 μM, the drug vehicle dimethylsulfoxide (DMSO) 1% and ethanol (EtOH) 70% at different incubation times, using the M. vogae TTy motility assay. These compounds were used as control in the anthelmintic effect determination of HDAC inhibitors. Relative motility indices were measured from three independent biological replicates, each one in quadruplicate. Error bars represent the standard deviation and the asterisks indicate those values that showed differences with statistical significance compared to the negative control (DMSO 1%), according to two-way ANOVA test and Dunnett’s post-tests (*, p < 0.05; **, p < 0.01; ***, p < 0.001; ****, p < 0.0001). (TIF) [file pntd.0009226.s005.tif]

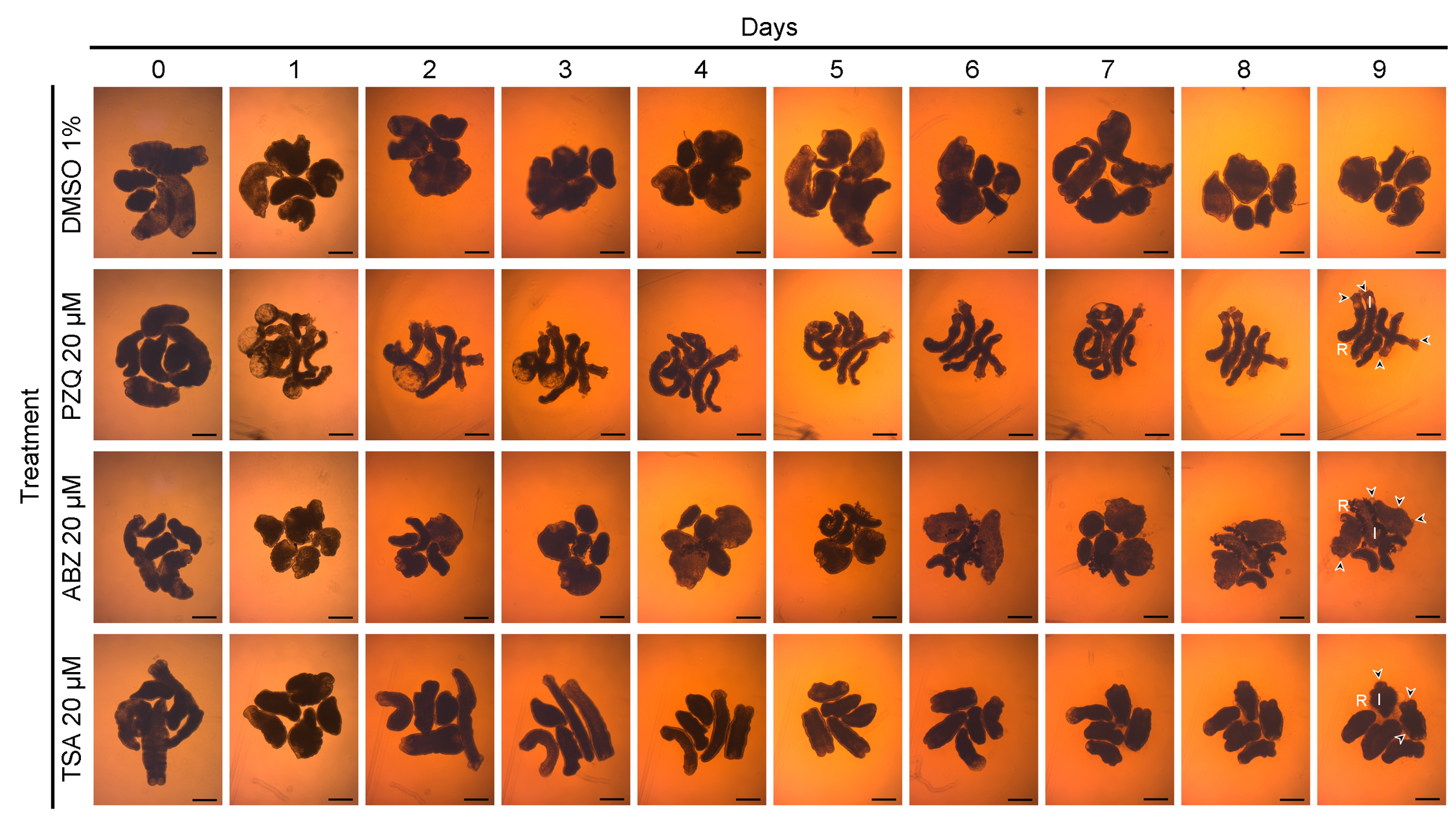

Supplement: S3 Fig — Inverted optical microscope images of M. vogae TTy treated with the current anthelmintic drugs, praziquantel (PZQ) and albendazole (ABZ), and the pan-HDAC inhibitor trichostatin A (TSA) at 20 μM at different days of treatment; compared to the parasites incubated with DMSO 1%. Note the extensive damage on the tegument with the presence of blebs (arrows) and loss of general parasite morphology, as well as the presence of influx (I) of culture medium into the worm and tegument debris (R) in the culture medium. These phenotypic alterations were observed for three independent biological replicates and were marked in the images at 9 days of treatment. Scale bars represent 100 μm. (TIF) [file pntd.0009226.s006.tif]

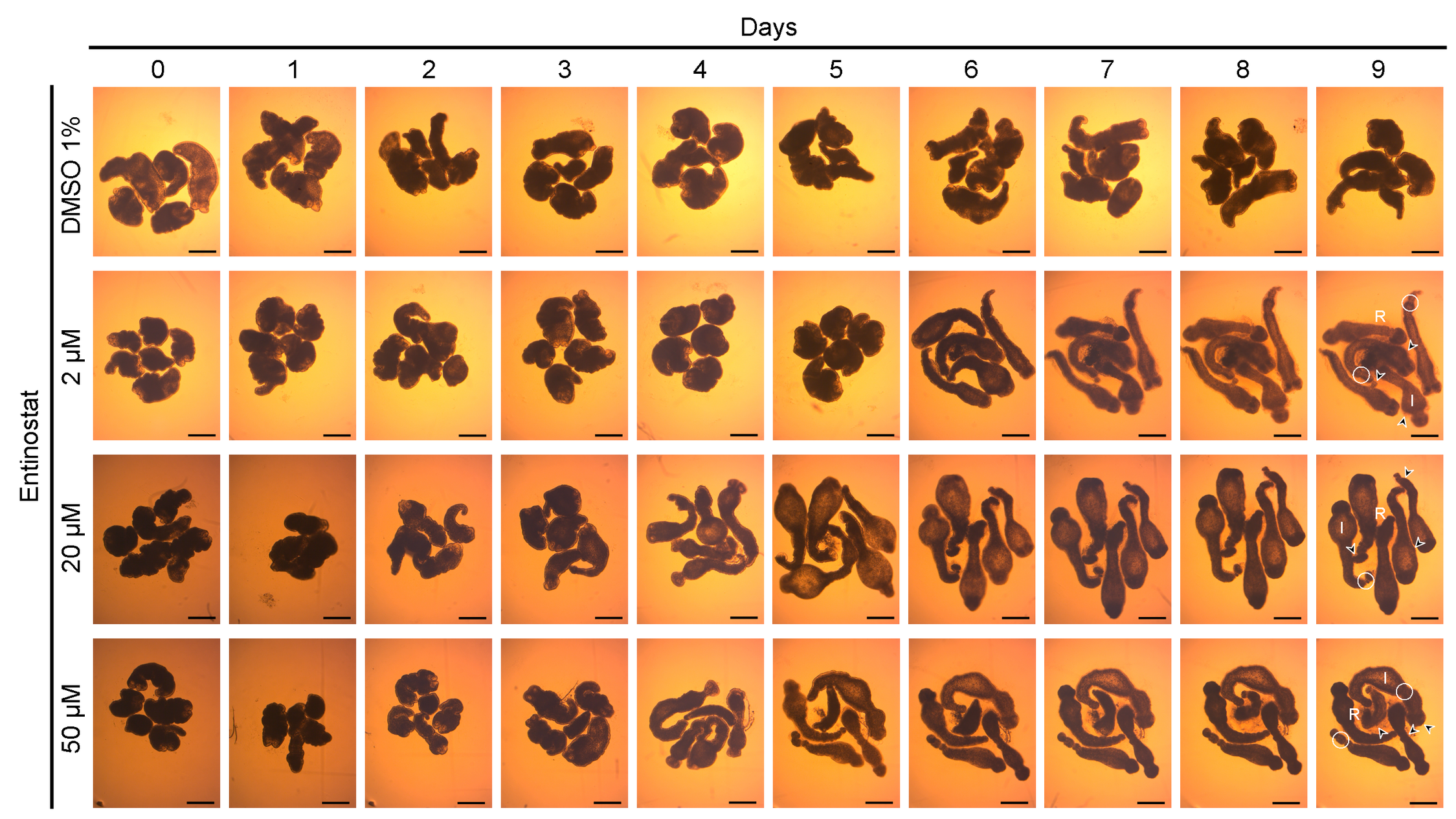

Supplement: S4 Fig — Inverted optical microscope images of M. vogae TTy treated with entinostat at 2, 20, and 50 μM and at different days of treatment; compared to the parasites incubated with DMSO 1%. Note the extensive damage on the tegument with the presence of blebs (arrows) and loss of general parasite morphology, as well as the presence of some constrictions on the body (circles), influx (I) of culture medium into the worm and tegument debris (R) in the culture medium. These phenotypic alterations were observed for three independent biological replicates and were marked in the images at 9 days of treatment. Scale bars represent 100 μm. (TIF) [file pntd.0009226.s007.tif]

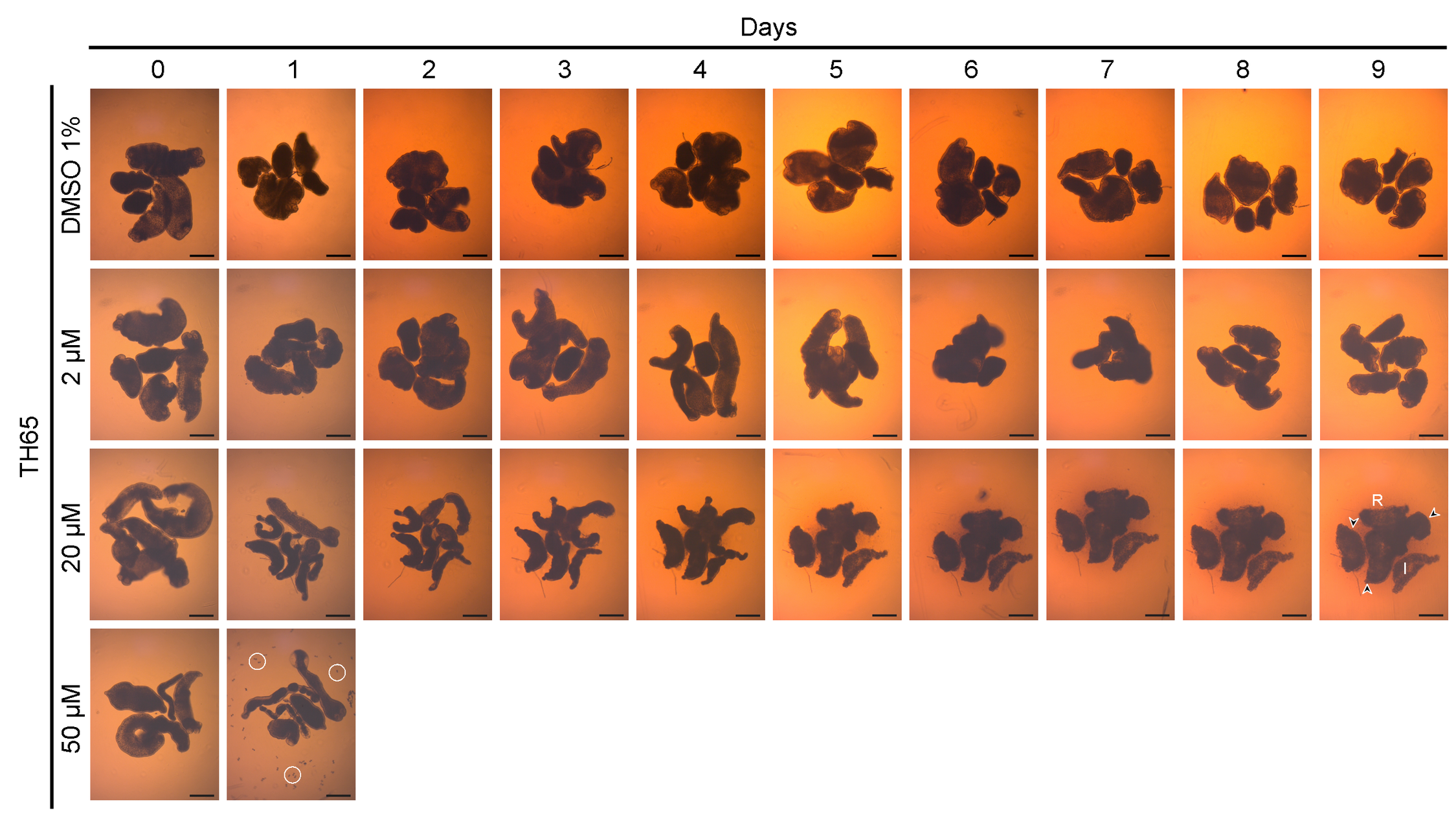

Supplement: S5 Fig — Inverted optical microscope images of M. vogae TTy treated with TH65 at 2, 20, and 50 μM and at different days of treatment; compared to the parasites incubated with DMSO 1%. Note the extensive damage on the tegument with the presence of blebs (arrows) and the complete loss of general parasite morphology, as well as the presence of the influx (I) of culture medium into the worm and tegument debris (R) in the culture medium. These phenotypic alterations were observed for three independent biological replicates and were marked in the images at 9 days of treatment. Additionally, note the formation of crystal-like structures in the culture medium whit TH65 at 50 μM after 1 day of treatment (marked with circles). Scale bars represent 100 μm. (TIF) [file pntd.0009226.s008.tif]

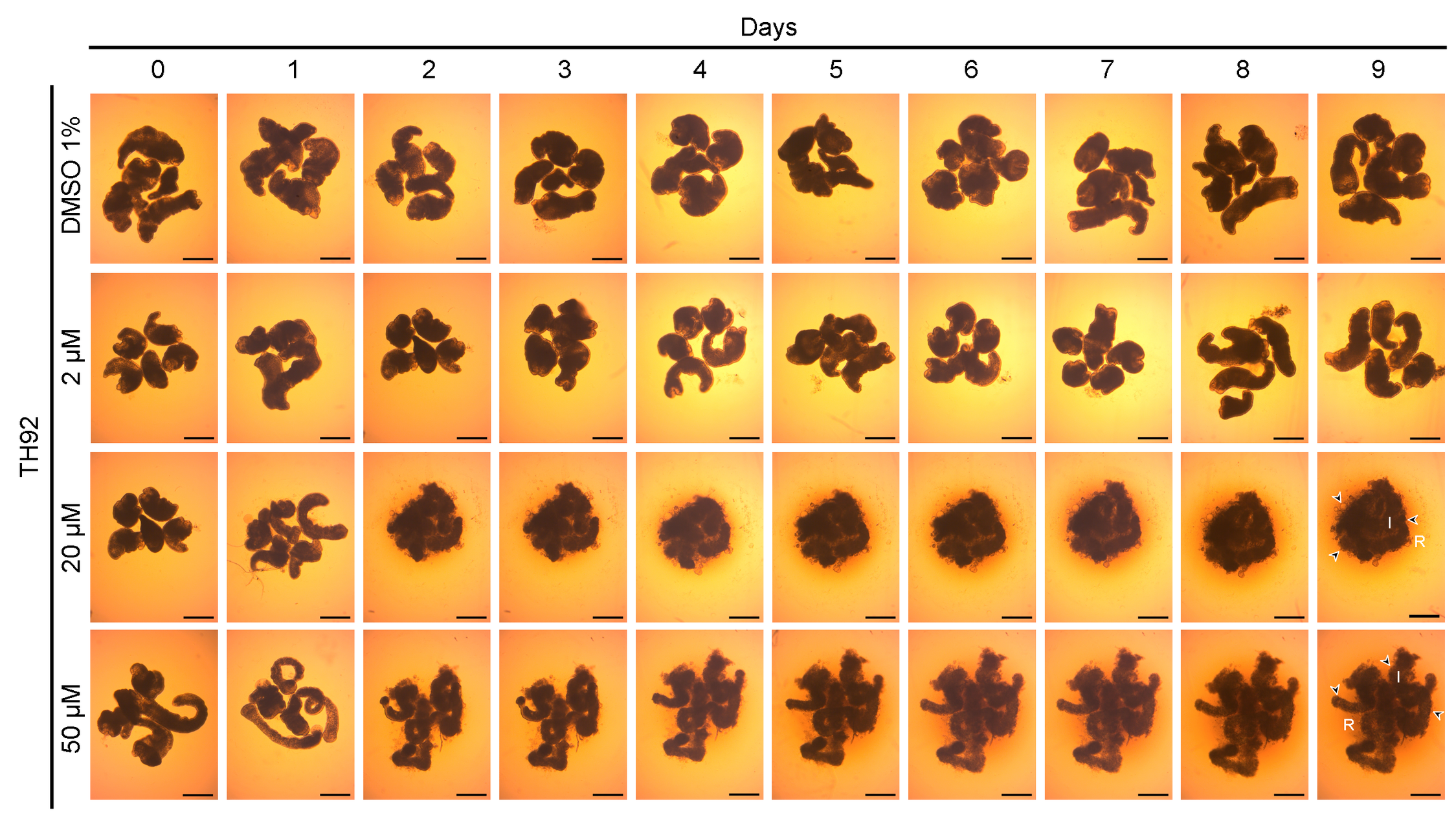

Supplement: S6 Fig — Inverted optical microscope images of M. vogae TTy treated with TH92 at 2, 20, and 50 μM and at different days of treatment; compared to the parasites incubated with DMSO 1%. Note the extensive damage on the tegument with the presence of blebs (arrows) and the complete loss of general parasite morphology, as well as the presence of the influx (I) of culture medium into the worm and tegument debris (R) in the culture medium. These phenotypic alterations were observed for three independent biological replicates and were marked in the images at 9 days of treatment. Scale bars represent 100 μm. (TIF) [file pntd.0009226.s009.tif]
